# Characterization and comparative analysis of microRNAs in the rice pest *Sogatella furcifera*

**DOI:** 10.1371/journal.pone.0204517

**Published:** 2018-09-24

**Authors:** Zhao-Xia Chang, Ibukun A. Akinyemi, Dong-Yang Guo, Qingfa Wu

**Affiliations:** 1 Hefei National Laboratory for Physical Sciences at Microscale, University of Science and Technology of China, Hefei, China; 2 CAS Key Laboratory of Innate Immunity and Chronic Disease, School of Life Sciences, University of Science and Technology of China, Hefei, China; Louisiana State University, UNITED STATES

## Abstract

MicroRNAs (miRNAs) are a class of endogenous regulatory RNA molecules 21-24 nucleotides in length that act as functional regulators of post-transcriptional repression of messenger RNA. We report the identification and characterization of a conserved miRNA and 171 novel miRNAs in the migratory rice pest *Sogatella furcifera* by deep sequencing, which were observed to be biased towards female adults of the insect, modulating the functionality and targets of the miRNAs in sex differentiation. A switch in arm usage was also observed in 9 miRNA when compared to the insect ancestor during insect evolution. The miRNA loci showed high 5’ fidelity in both miRNA and star species and about 93.4% of WBPH miRNAs conserved within non-planthopper species were homologous with planthopper species. The novel miRNAs identified in this study provide a better understanding of the sRNA and the regulatory role of miRNA in sexual dimorphism and alteration in the expression or function of miRNAs in the rice pest.

## Background

Among RNA types, microRNAs (miRNAs), small interfering RNAs (siRNAs) and Piwi-associated RNAs (piRNAs) are the best understood small RNAs (sRNAs) in the canonical RNAi pathway. Different classes of sRNA are defined by their sizes and interactions with the Argonaute (AGO) protein. In insect gene silencing, 21-24 nucleotide (nt) long miRNAs involved in post-transcriptional gene expression are generated by Dcr-1 and interact with AGO1 to form a RISC complex; the miRNA guides the RISC on its gene targets by sequence-specific base-paring to repress protein translation or destabilize mRNA transcripts [1–3]. Recently, miRNAs have been reported to be involved in regulating cell death and proliferation, fat metabolism and the differentiation of hematopoietic families in animal; they also regulate thousands of target genes in a complex network in humans [4]. Although knowledge of gene regulation via miRNAs had increased in recent years, there is still a limited understanding of the dynamic interactions between miRNAs and their targets beyond the down- and up-regulation of transcript levels of their targets binding with various genic sites [5, 6].

Next-generation sequencing has become the method of choice for annotating new miRNAs, including species-restricted genes, yielding great insights into miRNA biogenesis, AGO sorting, and post-transcriptional modification. Various insects such as *Drosophila melanogaster* [7], *Bombyx mori* [8], *Blattella germanica* [9], *Apis mellifera* [10] and *Aedes aegypti* [11] have also been reported to encode miRNAs that play important roles in development, adaptation and host-virus interactions. The analysis of small RNA libraries generated from various developmental stages in different species enables an in-depth study of miRNA in physiological processes [12]. In insects, including *D*. *melanogaster* and *Anopheles gambiae*, expression profiles and sex-biased miRNAs in sexual dimorphism have been characterized [1, 13, 14]. Several miRNAs are upregulated in the gonads during gametogenesis and play key roles in the reproduction and regulation of sex determination of *A*. *gambiae* and *D*. *melanogaster* [1, 14]. Consequently, miRNA plays a vital role in the regulation of genes involved in sex determination and maturation, as well as other biological processes for proliferation.

*Sogatella furcifera*, also known as white-backed planthopper (WBPH), is an important migratory rice pest of the family Delphacidae that results in the death of heavily infested plants in eastern Asia. WBPH and other planthoppers such as *Laodelphax striatellus* (LSTR) and *Nilaparvata lugens* (NLUG) also act as plant virus vectors against rice plants. Our previously sequenced genome of WBPH [15] provides a strong foundation for understanding the genetic and regulatory networks in WBPH [16], but little is known about the small RNA world involved in sex differentiation and determination in adult WBPH. In this study, six sRNA libraries of male and female WBPH adults were constructed and deep-sequenced. Analysis of the libraries revealed the functional organization of miRNA involved in regulatory expression, sex bias and identification of novel miRNAs expressed in adult WBPH. Conservation of miRNAs in WBPH, LSTR and NLUG in miRNA evolution has also enriched our understanding of the miRNA repertoire in WBPH. This study will provide new information on elucidating the regulatory role of miRNAs in sexually dimorphic traits and the development of vector control strategies for the insect pest.

## Method

### Sample preparation, RNA extraction and data processing

WBPH was reared and maintained to the adult stage (male and female) on healthy rice seedlings grown in a climate chamber with a temperature of 26°C, 70% relative humidity, and a photoperiod of 16 hours of light and 8 hours of dark [16]. Total RNA was extracted from male and female WBPH adults using TRIzol reagent (Life Technologies, U.S.A.) according to the manufacturer’s instructions. RNA quality and integrity were assessed using an Eppendorf BioPhotometer Plus (Germany). One microgram of total RNA was used to construct each small RNA library using a TruSeq Small RNA kit (Illumina, San Diego, CA, USA) and a previously described method [17] and sequenced on an Illumina HiSeq 2500 for the identification of miRNA from the samples.

Adaptor sequences were removed from the generated reads using the FASTX-toolkit (http://hannonlab.cshl.edu/fastx_toolkit/), and low-quality tags were cleaned to retain reads with length ≥ 18 and ≤ 30 nucleotides. The processed reads from each sample were mapped to the miRNA hairpin sequences [17] using Bowtie version 1.1.0, allowing for 0 mismatches. Reads mapping to snRNAs, snoRNAs, rRNAs, tRNAs, and known miRNAs were removed. The remaining processed reads from each sample were then mapped to the WBPH genome to filter out host sequences.

### Novel miRNA discovery

To predict novel miRNAs in the WBPH adults, we used the mireap software (http://sourceforge.net/projects/mireap/), maintaining default settings and filtering reads by size ≥ 18 nt. All clean reads were mapped to these pre-miRNA sequences using Bowtie version 1.1.0, allowing for zero mismatches. Non-repetitive loci, including exonic locations, were retained. Novel miRNAs required 10 or more mature strand reads and one or more star reads. The miRNA sequences were downloaded from the miRBase release 21 [18, 19] and searched against the WBPH genome [15] using BLASTn to identify potential miRNA homologs [18].

### Target prediction, functional annotation and enrichment analysis

Three different computational tools (MiRanda [20], PITA [21] and RNAhybrid [22]) were used to obtain reliable gene targets of miRNAs. The default parameters for each program were employed to predict the gene targets, except for the following specific parameters: (i) miRanda: score threshold (145), energy threshold (-10) kcal/mol [23]; (ii) PITA: seed region (length: 7–8, no mismatches, no G:U pairs); (iii) RNAhybrid: ∆G ≤ -20 kcal/mol. Gene enrichment in annotation terms (GO or KEGG) was measured using the Fisher exact test.

### miRNA homologs in NLUG and LSTR

The published genomes of *Laodelphax striatellus* (LSTR) and *Nilaparvata lugens* (NLUG) were downloaded via the GigaScience repository, GigaDB [24, 25]. All miRNA mature sequences were mapped to the NLUG and LSTR genomes by blastn (-W 7 -e 1). We extended 90 bp flanking the mapped location, and this location was assessed for hairpin structures using RNAshapes [26]. The maximal space between miRNA and miRNA* was 35 bp, and the maximal bulge of miRNA and miRNA* was 5.

### Differential expression and functional annotation

EdgeR was used to detect differentially expressed miRNAs between samples [27] and those with a p-value < 0.05 were deemed significant for downstream analysis. The assignment of Gene Ontology (GO) and Kyoto Encyclopedia of Genes and Genomes (KEGG) annotations for the gene sets was performed using InterProScan [28]. The false discovery rate (FDR) was calculated based on the method of Benjamini and Hochberg [29].

### RNA extraction, reverse transcription and real-time PCR analysis

Total RNA from *S*. *furcifera* adults (male and female) was isolated using TRIzol reagent and treated with Baseline‐ZERO DNase (Epicentre, Madison, WI, USA) to remove genomic DNA contamination before being used for cDNA synthesis. To quantify the miRNA, treated RNA (200 ng) was reverse-transcribed and quantified using the Bulge-Loop TM miRNA primer set (RiboBio, Guangzhou, China) for miRNAs versus U6 small nuclear RNA as a control. However, to quantify the putative miRNA target genes, treated RNA (200 ng) was reverse-transcribed using the mix random primer and oligo(dT), and quantified using gene-specific primers for the putative target genes of miRNA versus RP-L4 gene of WBPH as a control. The primer used for these assays are shown in S4 Table. Reverse transcription was performed using the RevertAid First Strand cDNA Synthesis Kit (Fermentas) according to the manufacturer’s instructions. Reverse transcription quantitative PCR (RT-qPCR) was conducted on a Light Cycler 96 (Roche) with FastStart Essential DNA Green Master mix. All reactions were performed in triplicate, and the expression level of RNAs was calculated using the 2−ΔΔCt method [30].

## Results

### Identification of miRNAs and their predicted targets in WBPH

In order to identify novel miRNA in WBPH, small RNA libraries were generated from male and female adults. A total of 114,975,733 reads were generated from 6 libraries that contain 60,713,943 and 54,261,790 reads of male and female adults, respectively (S1 Table). Reads mapping to snRNAs, snoRNAs, rRNAs, tRNAs, and known miRNAs were removed from the total reads, and the remaining unannotated reads were mapped against the WBPH genome to predict novel miRNAs in WBPH adults using mireap software (http://sourceforge.net/projects/mireap/). To identify the conserved miRNA, all clean reads were mapped to these pre-miRNA sequences, allowing for zero mismatches. We identified a conserved miRNA and 171 novel miRNAs with a read count ≥ 10 in the WBPH adults. These novel miRNA have a dominant read length of 22 nt, and the first nucleotide bias of novel miRNAs also showed a strong preference for ‘U’ at the 5’ end. Also, another conserved miRNA, mir-29 was identified with a BLAST search against the sequences deposited in the miRBase database (release 21) [18, 19]. As a whole, in addition to 106 conserved miRNAs and 276 novel miRNAs identified in our previous study of cultured cells [17] of WBPH, a conserved miRNA and 171 novel miRNA were identified in this study making a total of 107 conserved miRNA and 447 miRNA discovered in our study of WBPH (S2 Table).

To obtain reliable gene targets of miRNAs, we retained only the targets predicted by at least two programs for downstream analysis. Exactly 2,720 genes were predicted to be the targets of 171 novel miRNAs, and a total of 19,441 miRNA target pairs were obtained from the prediction. On average, each of these novel miRNAs has 113 targeting interactions, which were lower than the average target number (115) of conserved miRNAs. This result suggests that novel miRNAs in adults may be specific in function. The annotation of unique target genes of the novel miRNA compared with the Gene Ontology (GO) database provides useful information for understanding gene functions and specific processes that have occurred throughout the evolution of WBPH. The GO terms identified in our analysis for all of the putative target genes subjected to GO functional classification suggest that the functions of the genes targeted by the identified novel miRNAs are mainly associated with the oxidation-reduction process (GO:0055114), transmembrane transport (GO:0055085) and signal transduction (GO:0007165) (S1 Fig).

### miRNA expression in adults

**Fig 1:**
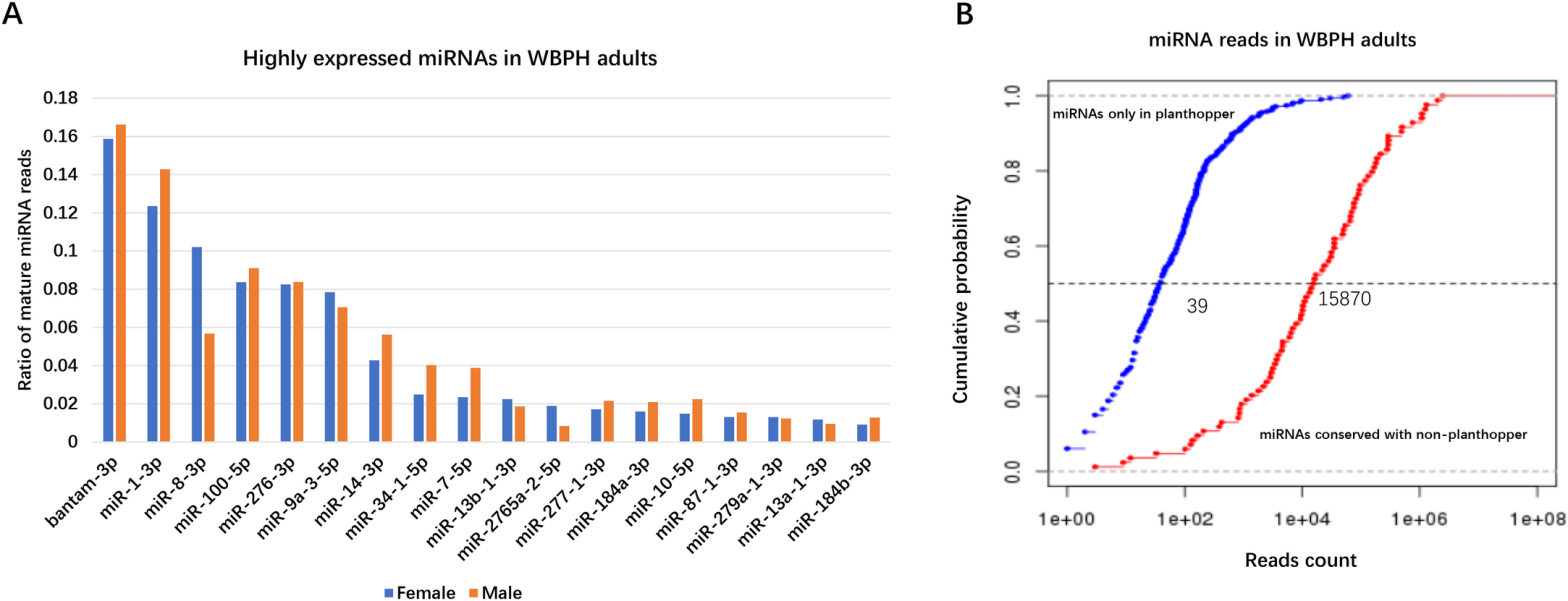
(A) Percentage distribution of highly expressed miRNA in WBPH adults (B) Reads count distribution of conserved and novel miRNAs.

In adults, the 18 top expressed miRNAs that contribute to more than 1% of the miRNAs were conserved and represent 87.3% of all the miRNA reads (Fig 1A). Bantam is the most expressed miRNA in WBPH adults. The high expression of WBPH bantam may be associated with the multi-functionality of the miRNA in both cells and adults, as discovered in *Drosophila* [31], which play various roles in germline stem cell (GSC) control, maintenance regulation, self-renewing division, and differentiation. However, several adult specific miRNAs were expressed in both sexes of WBPH, such as miR-1, which is known as muscle-tissue specific miRNA in adult mice [32]. Activation of TOR signaling in the thoracic muscles and signaling metabolites in tissue communication are affected by the regulation of branched-chain amino acid (BCAA) catabolism by miR-277 [33]. Furthermore, conserved miRNA reads counts were much more than novel miRNA reads counts (Fig 1B).

### Function shift in WBPH adults

**Fig 2:**
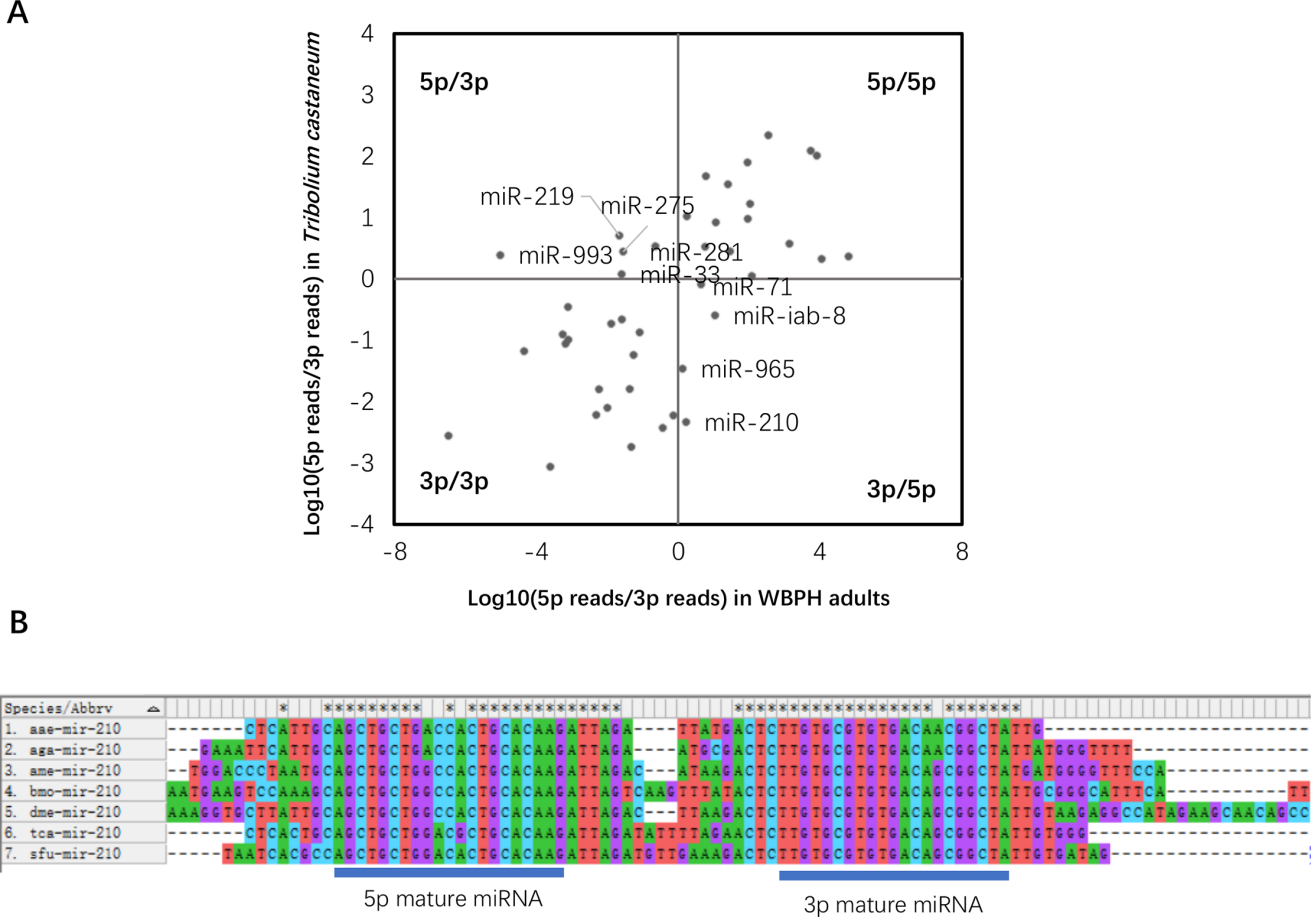
(A) Comparison of the arm usage bias between *Tribolium* and WBPH (B) conservation of miR-210 hairpin sequences in 7 insects. The abbreviation represents the species name. aae: Aedes aegypti; aga: Anopheles gambiae; ame: Apis mellifera;bmo: Bombyx mori; dme: Drosophila melanogaster tca: Tribolium castaneum.

Arm switching changes the predicted targeting preferences and alters the regulatory functions of miRNAs [34]. Sequences of the 5’ and 3’ arms of miRNA are different when opposite arm might regulate the expression of a different set of mRNA transcripts or common targets [35]. In *A*. *gambiae*, 20 miRNAs were identified in the tissue-specific expression of miRNA arm usage [36]. It was observed that changes in arm usage and selection of the identified miRNA in WBPH may affect or alter the functionality and targets of the miRNA. *Tribolium* miRNAs have been reported to be more conserved in vertebrates and are more representative of the insect ancestor than *Drosophila*[34]. We therefore compared the arm usage of *Tribolium* and WBPH (Fig 2A) using miRNA reads of *Tribolium* from a published study [34]. This clearly shows a switch in arm preferences of 9 miRNAs during insect evolution. For example, the 5’ arm of mir-281 produces the dominant product in *Tribolium*, whereas the 3’ arm dominates in WBPH. Switches were also observed in mir-219, mir-993, mir-33, miR-71, miR-iab-8, miR-965, miR-210 and mir-275.From the multiple sequence alignment analysis of hairpin structures in several species, the 3p and 5p ends sequences of miR-210 are relatively conserved, indicating that both ends can be used as stable miRNAs (Fig 2B). Expression of different WBPH miRNA transcripts or common targets can therefore be regulated by arm switching of the miRNA to provide a fundamental mechanism for the evolution of miRNA function.

### 5’ isomiRs of WBPH conserved miRNAs

**Fig 3:**
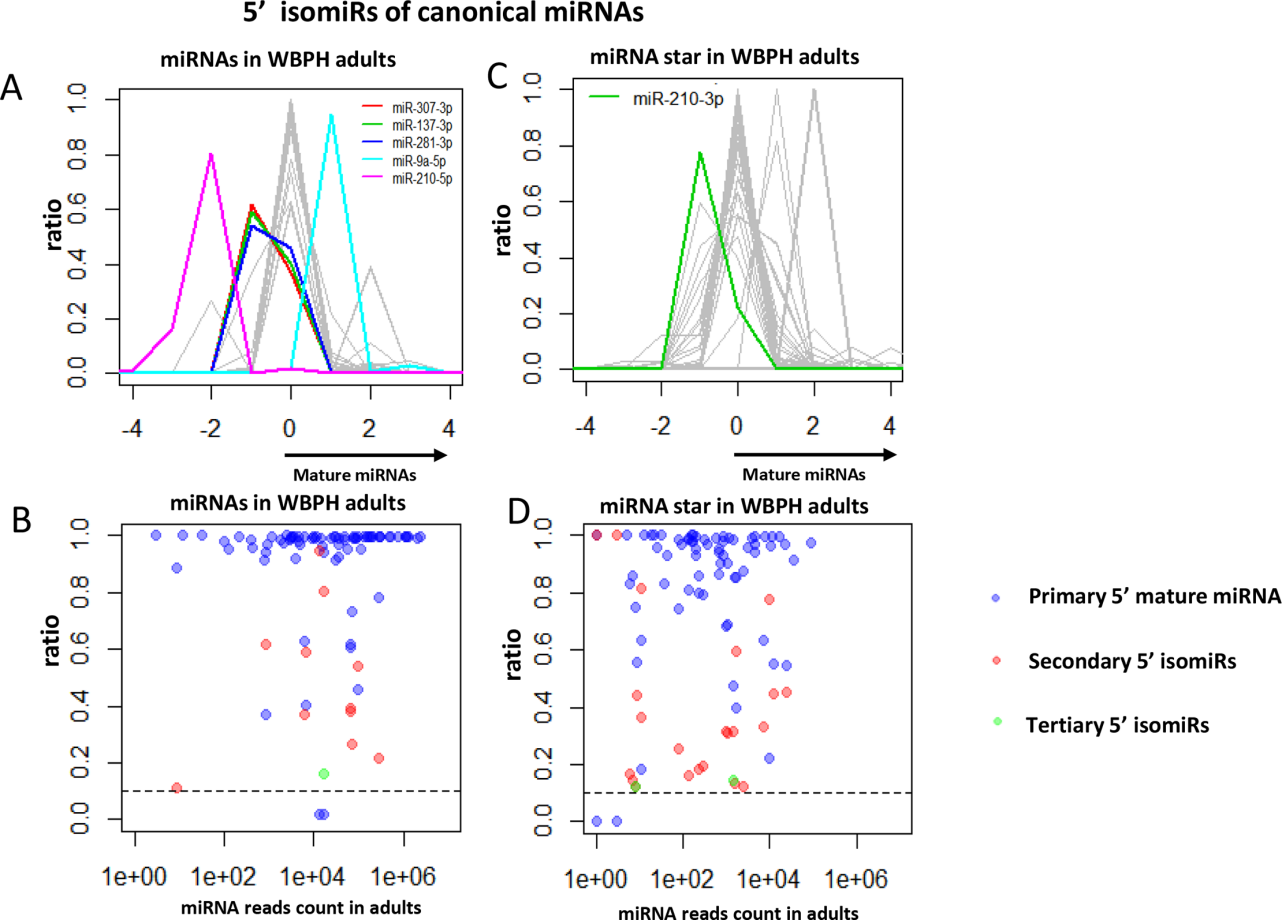
5’ variability of 76 conserved WBPH miRNAs. (A, B) 5’ end precision of mature miRNAs was higher than that of miRNA star species; (C, D) 5’ end precision of miRNA species in miRNA star was generally high for well-expressed species.

With respect to targeting common biological pathway functionalities of miRNA and heterogeneity or variants of most miRNA referred to as isomiRs [37, 38], we cataloged the 5’ variation of miRNAs of 76 conserved miRNAs. For conserved miRNA species, the 5’ end precision of mature miRNA species was generally higher than that of miRNA* (star) species (Fig 3). The majority of loci exhibited high 5’ fidelity of both miRNA and star species, as well as an asymmetric accumulation of miRNA and star species, such as mir-2796 (S2 Fig). A subset of highly imprecise 5’ ends of miRNAs and star species were clearly distinguished. Several loci generated abundant secondary and/or tertiary 5’ isomiRs, and highly expressed star species exhibited abundant 5’ isomiRs. Previously, the most striking case of a WBPH 5’ isomiR was miR-281, which exists as nearly equal populations of two different 5’ ends. In this case, the mature miRNA is produced from the 3’ arm, indicating heterogeneity at the Dicer cleavage step. mir-307 and mir-173 were another two loci with notable 5’ isomiR capacity on the mature 3’ strand.

### Sex-biased WBPH miRNAs

To determine which of the identified miRNA are sex-biased in their expression pattern, we compared libraries of adult female and male WBPH, respectively. Of all mature miRNA, 84 and 78 mature microRNAs showed a significant expression bias in males and females, respectively (S3 Table). We also investigated the potential roles of sex-biased miRNAs by performing target gene prediction. A total of 527 annotated genes were predicted to be putative targets of male-biased miRNAs, while 382 annotated genes were predicted to be putative targets of female-biased miRNAs. GO enrichment analysis indicated that putative male-biased miRNA target genes were mainly involved in protein phosphorylation, intracellular protein transport, and glutaminyl−tRNA aminoacylation. However, putative female-biased miRNA target genes were mainly involved in transmembrane transport and proteolysis.

**Fig 4:**
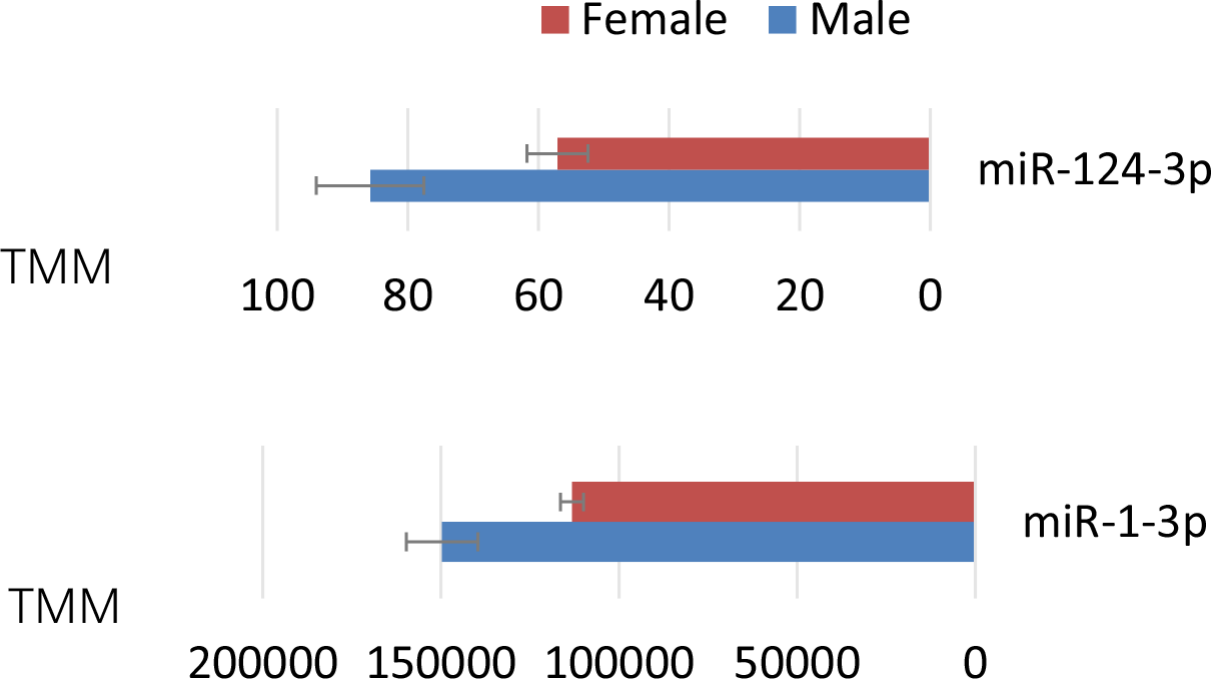
Sex-biased expression of WBPH miRNAs in male and female adult WBPH.

The results showed that miR-8-3p, miR-9a-5p, miR-9b-5p, miR-9c-5p, miR-n58-5p and miR-n174-5p were more highly expressed in female WBPH adults, however, miR-n58-5p and miR-n174-5p are closely located in the insect genome, forming a cluster, in which co-expressed miRNAs are processed from the same precursor RNA. The changes in the expression of miR-n174-5p were validated by qPCR using the same total RNA samples employed for the construction of the libraries. Previously, it was reported that miR-8 exhibits high levels of expression in the fat body of female mosquitos post-blood meal (PBM)[39] by acting on the wingless (Wg) signaling pathway and functions in the fat body of the female mosquito, which then affects reproductive processes [40]. The female-biased miR-9a/miR-9b cluster was found to be a conserved sex-biased miRNA cluster, while miR-9c of the same family is also female-biased in *D*. *melanogaster* [14]. However, miR-124-3p, miR-993-3p, miR-971-5p, miR-263a, miR-263b, miR-927-and miR-252a were preferentially expressed in the male WBPH adults. miR-124 in *Drosophila* is required for proper male-specific pheromone production [41]. In addition, miR-124-3p and miR-1-3p having higher expression in male adults (Fig 4A) probably glutamine—tRNA ligase (SFU-348.23, gln) and protein phosphatase (SFU-40.81). miR-124-3p can also target SFU-162.31(RNA-binding protein 26). For three of the putative target genes, RT-qPCR evaluation in both male and female adults shows that the expression levels of these target genes decreased in adult females (S3 Fig).

### WBPH miRNAs homologs in *Laodelphax striatellus* (LSTR) and *Nilaparvata lugens* (NLUG)

**Fig 5:**
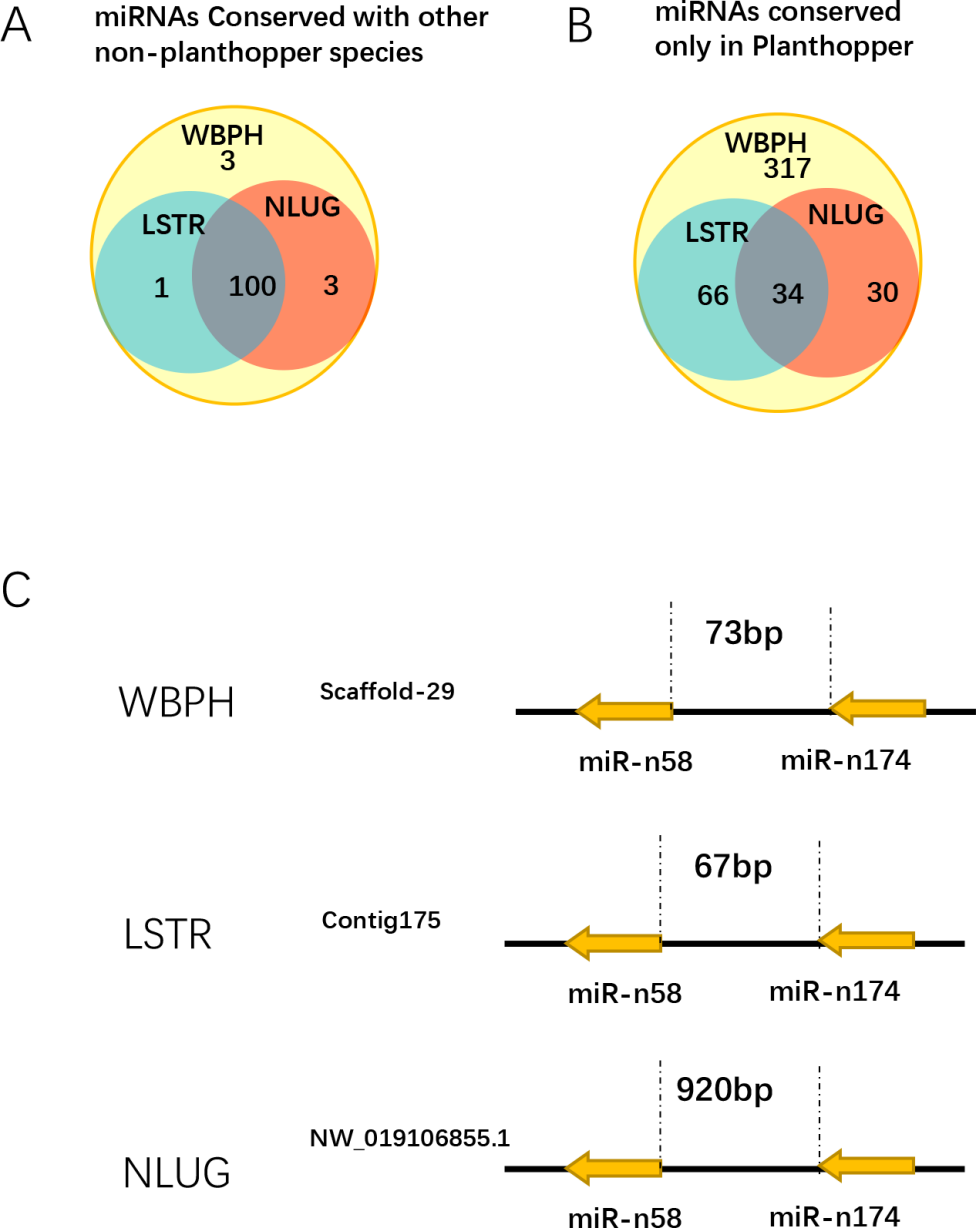
WBPH miRNA homologs in NLUG and LSTR. Homology prediction of identified WBPH miRNA in LSTR and NLUG from (A) non-planthopper and (B) planthopper species. (C) A WBPH miRNA conserved cluster.

We also conducted a homology-based search of the identified WBPH miRNAs in the recently published genomes of LSTR and NLUG [24, 25]. Exactly 93% (100/107) WBPH conserved miRNAs identified within non-planthopper spices were also found in both LSTR and NLUG. However, 7.6% (34/447) of specific WBPH miRNA were identified as homologous in both LSTR and NLUG by a genome-wide homology prediction (Fig 5A and 5B). These results suggested that most WBPH-specific miRNAs did not descend from their common ancestor and instead were *de novo* created during the evolution of the species after divergence from the two species to adapt to its lifestyle. In many species, miRNA analysis shows that a substantial fraction of miRNAs were genomically clustered, and these miRNAs in a cluster or family may have functional relationships by coordinately regulating biological processes [42, 43]. Twelve conserved miRNA clusters that are quite conservative in the planthoppers were identified in both WBPH and NLUG (Table 1). However, miR-92a/92b, which was found to be essential for the modulation of the PDF neuronal excitability and maintenance of a neuroblast pool in Drosophila [44, 45], was not identified in LSTR. In contrast, only two planthopper-specific miRNA clusters (miR-n58/n174 and miR-n113/n177) were found to be conserved in the planthopper. Interestingly, the two located near each other in the miR-n58/n174 cluster contain the same seed, suggesting that their appearance might occur through a replication event. In the genomes of WBPH and LSTR, the distances between the miRNAs in the miR-n58/n174 cluster are 73 bp and 67 bp, respectively, while in the genome of the NLUG, the distance between the two miRNAs is 920 bp, which is 13-fold larger than the clusters of WBPH and LSTR (Fig 5C). A sequence alignment of the NLUG miR-n58/n174 cluster to Repbase [46, 47] showed that 77 bp between the two miRNAs have 79% similarity with Polinton (also called Mavericks)—large DNA transposons that have gene homology to viral proteins and are often found in eukaryotic genomes. We hypothesize that during the evolution of NLUG, the transposon Polinton was inserted into the cluster and later “jumped” out of the nucleotide arrangements of the cluster with time and that the rapid evolution of the transposon led to increased distance between the two miRNAs within the cluster. Expression of miR-n58 and miR-n174 was extremely low in male WBPH adults but much higher in female WBPH adults. However, miR-n58-5p and miR-n174-5p have the same seed region, indicating they regulate similar target genes while miR-n174-5p target gene, SFU-17.151 (E3 ubiquitin-protein ligase RNF123) were up-regulated 1.74 fold in males (S3 Fig).

**Table 1:** Conserved miRNA clusters in WBPH, NLUG and LSTR.

Conserved miRNA clusters	WBPH	LSTR	NLUG
miR-306/9	✔	✔	✔
miR-279a/miR-279b	✔	✔	✔
miR-2/13/71	✔	✔	✔
miR-2788/193	✔	✔	✔
miR-283/747/12	✔	✔	✔
miR-1175/750	✔	✔	✔
miR-277/34	✔	✔	✔
miR-2765a/2765b	✔	✔	✔
miR-92a/92b	✔		✔
miR-275/305	✔	✔	✔
miR-100/let-7	✔	✔	✔
miR-9a/9b	✔	✔	✔
**Total**	**12**	**11**	**12**

## Discussion

Ever since the discovery of the first miRNA, characterization of small RNA has led to a better understanding of their functions as major post-transcriptional regulators of gene expression [3]. However, little is known about small RNA in WBPH and the biogenesis of the canonical pathway that may be responsible for sexual differentiation in the insect pest. To better understand the expression profiles of miRNAs in adult male and female WBPH, we identified and characterized the miRNA repertoire in both sexes using a deep sequencing approach. Specifically, a conserved miRNA and 171 novel miRNAs were identified in male and female adult WBPH, with various functions mostly expressed in sexual dimorphism.

The WBPH miRNA identified in this study shows that the expression of some miRNAs is sex-biased and that sex-biased gene expression between males and females of the same species might be responsible for phenotypic variation [48]. The expression patterns of most miRNA in the male adults of WBPH are differentially expressed compared to miRNAs in female adults. Biological processes in insects are affected by different miRNA expression patterns experienced in different insect sexes, which have been suggested to play a key role in gonad differentiation. Moreover, the identification and characterization of sex-biased miRNA in WBPH adults have important implications for phenotypic variation between males and females [49], with some highly expressed gender-specific miRNAs in WBPH. Specifically, the target gene prediction revealed that miRNA expression identified in this study is more biased towards females than males, which may also be important for sex differentiation. The miRNA profiles of both male and female WBPH described in this study will increase our understanding and provide better resources for the regulation of genes for effective vector control.

A homologous search of WBPH miRNA in the NLUG and LSTR genomes revealed that 34 WBPH miRNAs are conserved among them, which suggest that these miRNAs have been under strong selective constraint throughout the evolution of planthoppers, as well as a convergent regulatory regime between these species. The mechanisms of miRNA origination and evolution promote the production of paralogous members of identical or nearly identical mature sequences, many of which are clustered and expressed in a cell-specific or tissue-enriched basis in different species [50]. In plants and animals, miRNAs are frequently clustered and can be independently or simultaneously transcribed into single polycistronic transcript through tandem duplication [51, 52]. One planthopper-specific cluster, miR-n58/n174, had exactly the same seed sequences suggesting that the origin of this miRNA cluster-forming event is a tandem duplication. However, after various evolutionary events, the copies in the cluster may be subject to sequence divergence and subsequent neofunctionalization. In addition, the longer miRNA distance of the NLUG genome in contrast to WBPH and LSTR indicates the insertion transposon in an evolution event aimed at splitting the cluster and moving one of the miRNAs to a more distant transcription unit. Interestingly, the miR-n58/n174 cluster expression level tends to be highly expressed in WBPH females but the miR-n174 target, SFU-17.151 (E3 ubiquitin-protein ligase RNF123), is down-regulated in female WBPH adults, indicating an important function in female adults.

Overall, the identified and characterized novel miRNA in male and female adults and the predicted target genes in this study provide further in-depth understanding of the sRNA in WBPH, especially for miRNA that are biased in sex determination. A comparative analysis of the expression and conservation of WBPH miRNAs with other planthoppers (NLUG and LSTR) also revealed the evolutionary functionality of the miRNA, which will serve as an important resource for further investigation into the role of WBPH miRNAs during different developmental stages and sex differentiation which can be used for the development of effective control strategies for the insect pest.

## Supporting information

S1 Fig **Novel miRNA (a) GO and (b) KEGG enrichment analysis (top 15 terms)**.
(PDF)

S2 Fig miR-2796 5’ isomiRs in WBPH adults. (PDF)

S3 Fig Sex-bias miRNA target gene expression in male and female WBPH. (PDF)

S1 Table Sequence reads generated from each library of adult male and female WBPH in triplicate. (PDF)

S2 Table Summary of miRNAs identified and characterized in WBPH. (PDF)

S3 Table Differentially expressed miRNA in male and female adults. (PDF)

S4 Table Primers used in RT-qPCR for validation of the miRNA genes. (PDF)
