# Supplementary figures and images for: Characterization and comparative analysis of microRNAs in the rice pest Sogatella furcifera
Source: PLoS One. 2018 Sep 24;13(9):e0204517. doi: 10.1371/journal.pone.0204517 (PMC6152972; doi:10.1371/journal.pone.0204517)

a

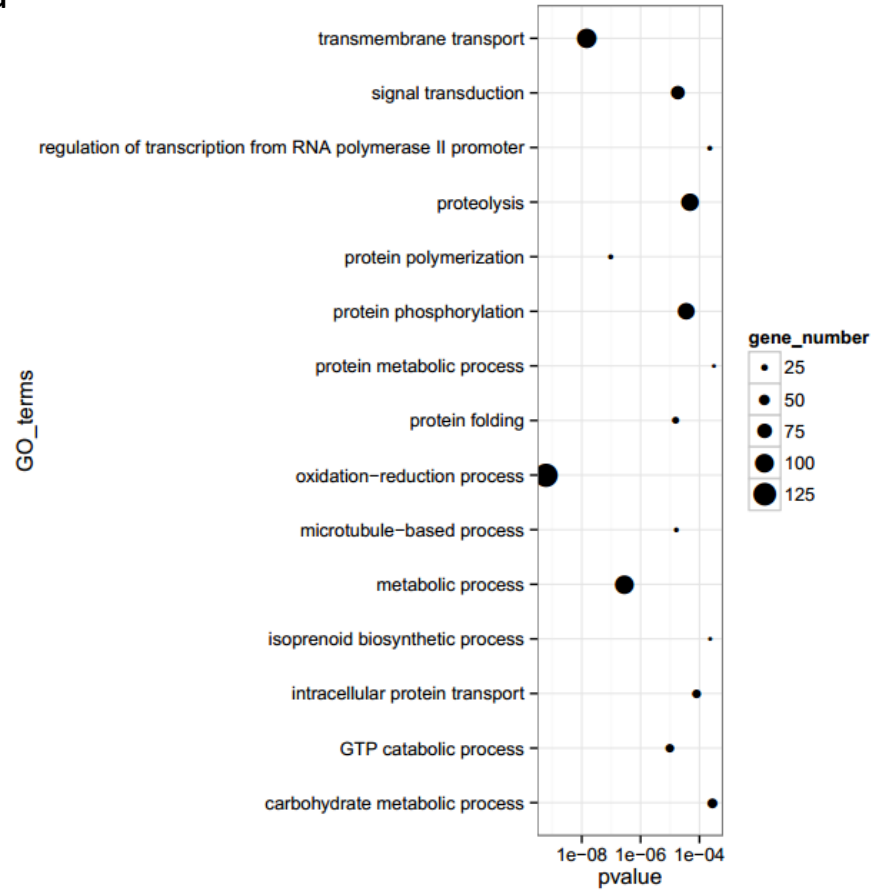

b

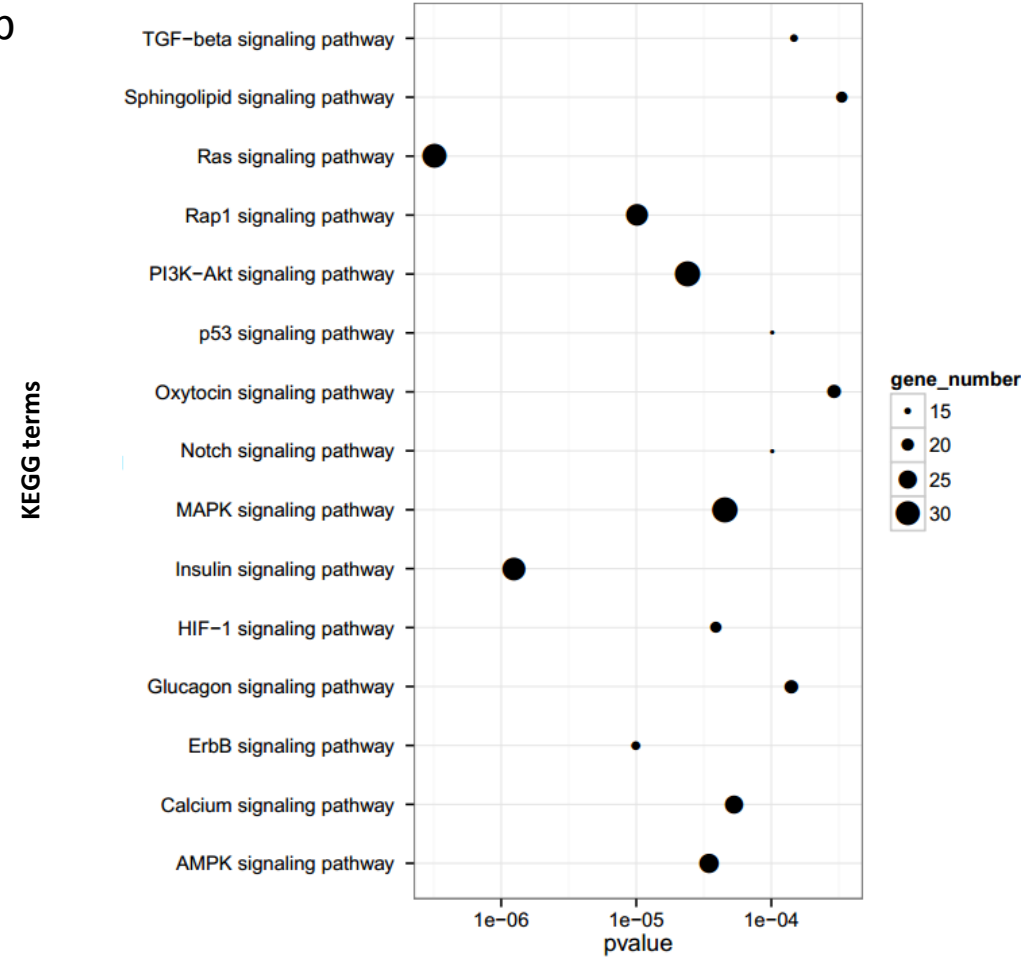

**S1 Fig.** Novel miRNA (a) GO and (b) KEGG enrichment analysis (top 15 terms)

Supplement: S1 Fig — Novel miRNA (a) GO and (b) KEGG enrichment analysis (top 15 terms). (PDF) [file pone.0204517.s001.pdf]

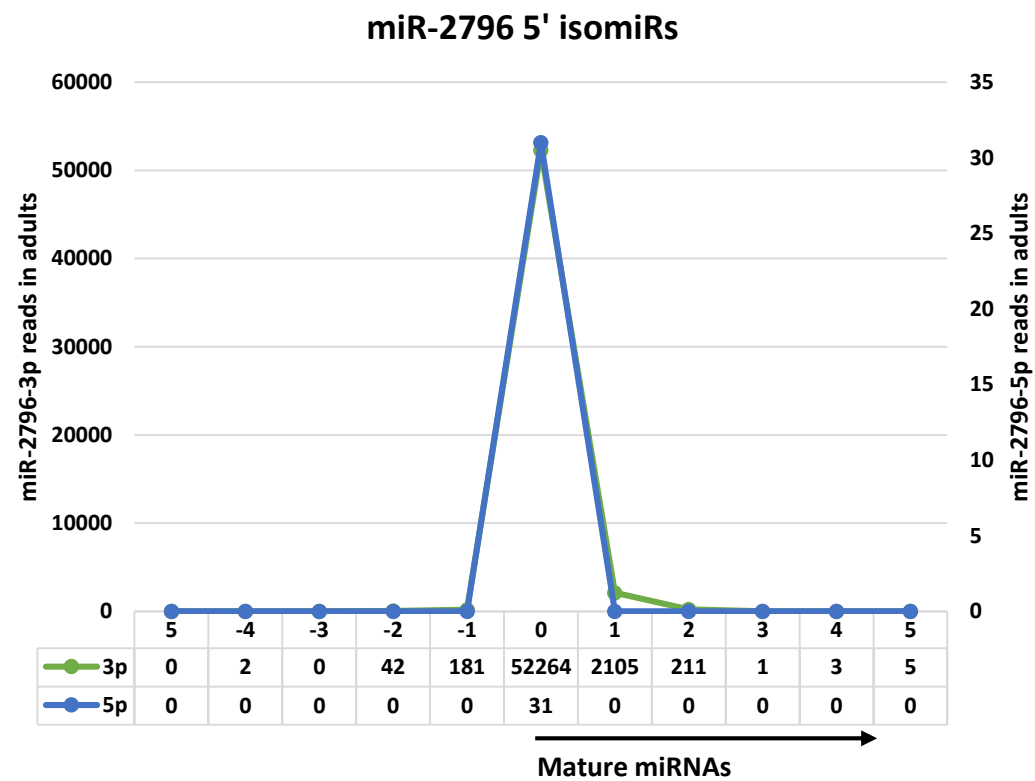

**S2 Fig.** miR-2796 5' isomiRs in WBPH adults

Supplement: S2 Fig — (PDF) [file pone.0204517.s002.pdf]

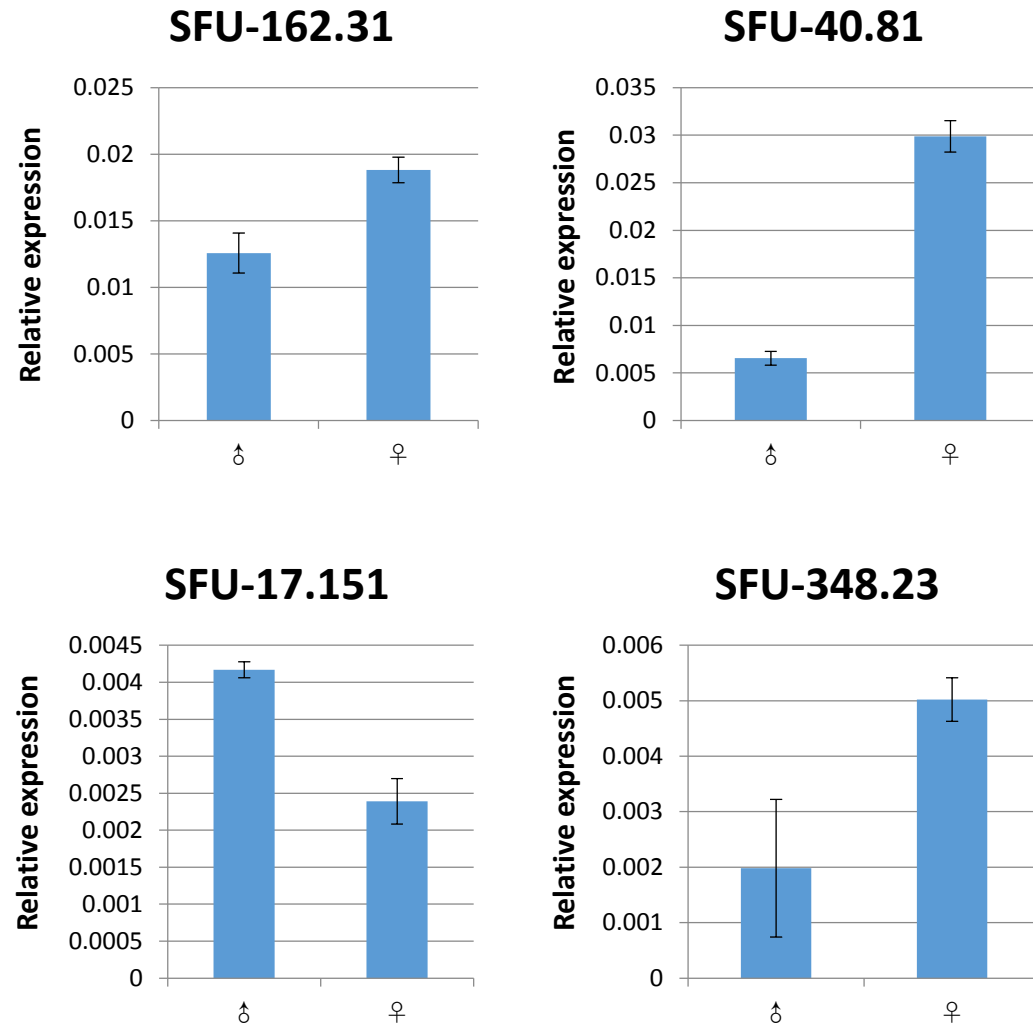

**S3 Fig.** Sex-bias miRNA target gene expression in male and female WBPH

Supplement: S3 Fig — (PDF) [file pone.0204517.s003.pdf]
