# Supplementary material for: Characterization and comparative analysis of microRNAs in the rice pest Sogatella furcifera
Source: PLoS One. 2018 Sep 24;13(9):e0204517. doi: 10.1371/journal.pone.0204517 (PMC6152972; doi:10.1371/journal.pone.0204517)
Supplement: S1 Table — (PDF) [file pone.0204517.s004.pdf]

S1 Table. Sequence reads generated from each library of adult male and female WBPH in triplicate

Reads(♂)	Maa	Mbb	Mcc
**total RNA**	22717122 (100.0%)	20003415(100.0%)	17993406(100.0%)
**rRNA**	1174311 (5.2%)	934660(4.7%)	1386805(7.7%)
**tRNA**	1441543(6.3%)	1169614(5.8%)	957660(5.3%)
**other sRNA**	32856(0.1%)	93527(0.5%)	30187(0.2%)
**snRNA**	38267 (0.2%)	28779(0.1%)	30370(0.2%)
**Reads(♀)**	**Faa**	**Fbb**	**Fcc**
**total RNA**	18922519(100.0%)	18192509(100.0%)	17146762(100.0%)
**rRNA**	625421(3.3%)	874098(4.8%)	706590(4.1%)
**tRNA**	722017(3.8%)	492988(2.7%)	510249(3.0%)
**other sRNA**	12203(0.1%)	16462(0.1%)	11571(0.1%)
**snRNA**	21902(0.1%)	25799(0.1%)	21788(0.1%)

Maa-Male adult, **Faa**- Female adult
