# Supplementary material for: Characterization and comparative analysis of microRNAs in the rice pest Sogatella furcifera
Source: PLoS One. 2018 Sep 24;13(9):e0204517. doi: 10.1371/journal.pone.0204517 (PMC6152972; doi:10.1371/journal.pone.0204517)
Supplement: S2 Table — (PDF) [file pone.0204517.s005.pdf]

**S2 Table.** Summary of miRNAs identified and characterized in WBPH

| miRNA | 5p miRNA sequence | 3p miRNA sequence | genome | hairpin site | strand |
|---|---|---|---|---|---|
| miR-n76 | GGCATCAAAGATGAGATGGAC | ATCTCATCTTTGATGTCCACTGC | Scaffold-142 | 810773 | - |
| miR-2c-1 | TCATCAAAGCCGGATTGTCATA | TCACAGCCAGCTTTGATGAGCAC | Scaffold-287 | 308637 | - |
| miR-n56a-1 | TGGTAAGTTCCACCACTATCTCA | AGATAGGGTGGTTGTTACCATTC | Scaffold-215 | 690053 | - |
| miR-n143 | CAAGGTCATAATGGGATGAATT | TTTACCCTTTTATGGCCTTGCGA | Scaffold-43 | 1996233 | + |
| miR-n91-2 | CATATTTATCATTGTATTGTA | TGCAATCCATTGAATAAATACGAA | Scaffold-432 | 193303 | + |
| miR-n149-2 | TAGGGAAGGACTTGCTTCAATCC | ATTAAGTTCGTCTTTTCCTGCT | Scaffold-215 | 774272 | - |
| miR-n73b | GCTGGGACTCTTGGGCTTTATG | TGAAGCTCATTAGCCCCAGTGT | Scaffold-24 | 86446 | + |
| miR-n90b-5 | TGTATTCACCTTTTAGCACGTTCA | TGTGATGTGAGGTGAGTGCAAT | Scaffold-215 | 599160 | - |
| miR-n82a | GGGTAGTGTTTCTGATATTATC | TAATATCATTGACACTACTTTT | Scaffold-160 | 163273 | + |
| miR-n98a-3 | AGGAAAGAGAAAGAGGGCAAAT | TTGATCATTTTCTCTTTTCTGT | Scaffold-1740 | 1281 | + |
| miR-n127-1 | TGAGGCCATGCAGAGCTGAAT | TTTGGTTCCTCATGGCCTCATGAT | Scaffold-896 | 12551 | - |
| miR-n199 | CGTGATGTATCATAGGCTTGTTG | ACAGTCTCTGATATATCACGTAT | Scaffold-160 | 173845 | + |
| miR-n95-1 | ACTGAGGCTCTTGCGGTGAGATG | TCTACTACATTAGCCTCAGTGT | Scaffold-71 | 139063 | + |
| miR-n47 | AGATGTCACTTCACCCTAATATGC | AGTTGGGATGTAATGACATC | Scaffold-15 | 4270046 | - |
| miR-n161 | TACCCTCAAAACTTTTAAAAAA | TTTTAAAAGTTTTTAGGGTATT | Scaffold-7 | 3544044 | - |
| miR-n57b | TGAGATCATAATGAAAGTTAT | TGGGCTTCCAGTATGATCTGT | Scaffold-215 | 575673 | + |
| miR-747-1 | TAATCTCATGTGGTAATGATACA | TATCATTATCAAATGGGATT | Scaffold-57 | 35414 | - |
| miR-n116 | AACGTTTGGGGTTGTTTCAAGA | TTTAACACATCTCAAACGTCGA | Scaffold-15 | 3611753 | + |
| miR-n4 | TGTATTTCAAGATCTGCCAAAG | TTGGCAGAGATTGAACGGAGCAAT | Scaffold-283 | 364135 | - |
| miR-n128-2 | TGAGGAACATTTTCTGGTCATA | TGACTAGATTCTGTTCCTCAGA | Scaffold-215 | 558155 | + |
| miR-n114-2 | CTTGGGAGGGATGTGTTGTATGT | TATTACACACCTCTTCCAAGATA | Scaffold-24 | 120710 | + |
| miR-n196a-1 | CGAGGAACATTTTCTGGTCAT | TGACTAGATACTGTTCCTCAGA | Scaffold-1096 | 17759 | + |
| miR-n55 | TGGTAACTCCCACATCAATGTCA | AAATTGTGTGATAGTTACCATTCT | Scaffold-215 | 411143 | + |

| | | | | | |
|---|---|---|---|---|---|
| miR-276 | AGCGAGGTATAGAGTTCCTACG | TAGGAACTTCATACCGTGCTT | Scaffold-34 | 1379393 | - |
| miR-n86 | GGGCTTTTCATTGGGCGGCTTTACC | CAGCTGCCCTATGAAGAGCAAC | Scaffold-202 | 67938 | - |
| miR-n195a-8 | AGCTGGCATACTTGTTTCCTATG | TAGGAACAATTATGCCGGTTTT | Scaffold-542 | 195438 | - |
| miR-n131-1 | CAATATCAGCCGGTGATTGCGA | GAGATTACCTGCTGATATTTAGG | Scaffold-312 | 10254 | + |
| miR-n3-3 | ATGGAGTCCGAAGTCACAGAACATT | TTCATGTGGCGGAATTGACTTCA | Scaffold-1622 | 17247 | + |
| miR-n101 | GCTGAGAAGAGACGTCGTCACAA | TGGGCGGAGTATCTTCTCGGCGT | Scaffold-67 | 2033166 | - |
| miR-n198b-1 | TGAGGTCATGCAGAACTAAATT | TTTGGTTCCTCATGGCCTCACGA | Scaffold-215 | 542236 | + |
| miR-n187 | AGCTGGTATGATTTTGCTAAGG | TTTGCATTATCATGCTAAGCTAG | Scaffold-24 | 3667680 | + |
| miR-n100-3 | TGTTGTTGGGAGACCAAAAATGT | TAATTTGGTCTACTCAACAGCACA | Scaffold-24 | 130209 | + |
| miR-n154 | AGAAGACACGTCGTTACAATGTA | TGAACGGAGTGTCTTCTCAGCT | Scaffold-32 | 1332450 | + |
| miR-n201a | TGGGGAACATTATCTGGTCATA | TGAGCGGATTCTATTCTCTAGT | Scaffold-43 | 1976783 | + |
| miR-n63b-5 | TGTTGTTGGGAGACATGAAATG | TTTCATGTCTATGCCCAACAGCA | Scaffold-542 | 182228 | - |
| miR-n81a-2 | TGATGGAAGGGAGTTCCTCAAT | TGTGGAGCTCCTCTTCCATCAGA | Scaffold-15 | 7269439 | + |
| miR-n67-1 | AGTAATTATCGCGTCAATCT | AGATTGTGTGATAGTTACCATTCA | Scaffold-215 | 444157 | + |
| miR-n142-1 | AATCTCATAAGGTAATGATATA | TATCATTATCGAATGGGGTTTCA | Scaffold-312 | 12631 | + |
| miR-981 | TCGGGTTTCTTGACAATGGACC | TTCGTTGTCGACGAAACTCAGAA | Scaffold-70 | 428404 | - |
| miR-193-2 | TCGGGACTTGGTGGTATAGTTGG | TACTGGCCTGCTAAGTCCCAAG | Scaffold-232 | 877826 | + |
| miR-n195a-3 | AGCTGGCATACTTGTTTCCTATG | TAGGAACAATTATGCCGGTTTT | Scaffold-15 | 7267578 | + |
| miR-n107d-1 | TGAGGTCTCGCAGAACTGAATT | TTCAGTCTGCAAAACCTCAAGA | Scaffold-215 | 649221 | - |
| miR-n192c-2 | TGGCAATGCAGGAGCTTTAATTG | ATAAAGCTTTTGCACTGTCAGA | Scaffold-1436 | 24897 | + |
| miR-n207 | TGGGTATATATATGGGAGGT | AGAGCTATCATTGTGTTCCTGACA | Scaffold-935 | 8442 | + |
| miR-n77a | TACAGAAAGTAGAATTCAGATGA | ATTTGCTTTCTTCTTTCTTGCT | Scaffold-7 | 1432964 | - |
| miR-13a-3 | CGTCAAATTGGTTGTGAGTTATG | TATCACAGCCACTTTGATGAAC | Scaffold-4365 | 2334 | - |
| miR-n78b | AGTGGGACAGTTCAAGATTGTTG | CCAGCCTTGGCTTTCCCACTGTAG | Scaffold-1740 | 11119 | + |
| miR-n105b-2 | CTTCTCATGATCCCGGTGATAC | TACACTGGGATTATGAGATTTA | Scaffold-325 | 77980 | + |

| | | | | | |
|---|---|---|---|---|---|
| miR-n138 | TGAGGTCATGCAGAACTAAATTC | TTTGGTTCCTCATGGCCTTACGA | Scaffold-215 | 671528 | - |
| miR-n194h | TAGTATGGCGGGTTCTTCTCA | TGAGGAGTTCCCCCATATTCAT | Scaffold-542 | 193054 | - |
| miR-13b-2 | ACGTCAAAATGGTTGTGAAATG | TATCACAGCCATTTTTGACGTACC | Scaffold-402 | 384508 | - |
| miR-n106 | TTCCGTCCAGCCTTCAAGAGTCAGG | GACTTGGAGGCAGAACTGAACG | Scaffold-20 | 5123133 | - |
| miR-2765c-2 | TGGTAACTCCACCACCGTTGGC | CAATGGTGCTGAAGTTCCTACA | Scaffold-1862 | 14441 | - |
| miR-n68 | TAGCGAATCTGAAGTTTGTTCT | TTTCAAATTTGTTACTCCTAGTT | Scaffold-249 | 264511 | - |
| miR-n130-2 | TGAGGAACACTGACTAGTCATT | TGGCTAGTTTTTGTTTCTCATA | Scaffold-215 | 774110 | - |
| miR-n202a-1 | TTTAAAGGGGCCTCACAGTAACG | TTACTGTGCGGCTACTTTATCT | Scaffold-1096 | 18103 | + |
| miR-n158 | TAGCGAATGTCCATTTCG | TGCGACGTTCACATTCGTTTCT | Scaffold-62 | 3278621 | + |
| miR-n32a-2 | GAAGTCATCTCAGCATTATATGC | AAAGATGCTGTTATGACTTCACT | Scaffold-24 | 125362 | + |
| miR-n185-2 | CGTGGCGCCTCAGGTATTAATT | TTAATATCTGTGGGGCTGCAAT | Scaffold-215 | 563363 | + |
| miR-9a-1 | TCTTTGGTTATGTAGCTGTATGA | ATAAAGCTATATTACCAAAGCA | Scaffold-1084 | 9760 | + |
| miR-929 | AAATTGACTCTAGTAGGGAGT | CTCCCTAATGGAGTCAGATTGAT | Scaffold-264 | 39185 | + |
| miR-n194b-4 | TAGTATGGAGGGCTCTTCTCAAT | TGAGGAGTTCCCCCATATTCAT | Scaffold-15 | 7259868 | + |
| miR-n84-2 | TGGCAATTTTCACATCAATCTCA | CGATTGTGTGATTGTTGCCATT | Scaffold-160 | 222554 | - |
| miR-n194k | TAGTATGGCGGGTTCTTCTCA | TGAGGAGTTCCCTCCATTCAGA | Scaffold-15 | 7259711 | + |
| miR-n144a-1 | TGTTGGAGACGTGATTAATTATA | TATTAATCTCGTTTGCACCAG | Scaffold-15 | 7269125 | + |
| miR-n133 | TGAGGCATGGTGGAAATAAGAATA | TTCATCTCTCCCATGCCTTGCC | Scaffold-542 | 181965 | - |
| miR-n107a-3 | TGAGGTCTCGCAGTACTAAATT | TTTAGTCTGCAAAACCTCAAGATA | Scaffold-215 | 653481 | - |
| miR-n5 | TACAAAATCTTCTGGATAGAACT | CTACCAGTAGAATTTGTAAGGC | Scaffold-313 | 262534 | - |
| miR-n43b | TGAGATCATCATGTTTATTACT | TAATCTCATGATTGATCTCAAG | Scaffold-160 | 262219 | + |
| miR-n127-5 | TGAGGCCATGCAGAGCTGAAT | TTTGGTTCCTCATGGCCTCATGAT | Scaffold-215 | 725784 | - |
| miR-n149-1 | TAGGGAAGGACTTGCTTCAATCC | ATTAAGTTCGTCTTTTCCTGCT | Scaffold-215 | 410369 | + |
| miR-n52b-2 | CAGGTACTATGTTTGATATTGTC | TAATATCATTCACAGTACTGCT | Scaffold-160 | 262420 | + |
| miR-219 | TGATTGTCCAAACGCAATTCT | AGAAATGCGTGTGGACATCAAC | Scaffold-302 | 401237 | + |

| | | | | | |
|---|---|---|---|---|---|
| miR-932 | TCAATTCCGTAGTGCATTGCAGTG | TGCAAGCAGTGCGGAGTTGTGCC | Scaffold-563 | 1000 | - |
| miR-n127-3 | TGAGGTCATGCAGAGCTGAAT | TTTGGTTCCTCATGGCCTCATGAT | Scaffold-215 | 594947 | - |
| miR-n39-1 | AAGTTTTAGTACATCATCATGCGAC | TTGGCATGGAATCTGGAATCATTGA | Scaffold-2067 | 16422 | - |
| miR-n201b | TGGGGAACATTATCTGGTCATA | TGAGCGGATACTATTCTCTAGT | Scaffold-43 | 1994893 | + |
| miR-n160 | AGTCCAATGCACACCAAAGTCT | TCTTTGGTATGTATTGGTCT | Scaffold-19 | 803848 | + |
| miR-87-1 | GGGCCTGAATCCTTACTCAACCT | GTGAGCAAAGTTTCAGGTGTGT | Scaffold-42 | 930408 | + |
| miR-n52a-4 | CAGGTACCGTGTTTGATATTGTC | TAATATCATTCACGGTACTGCT | Scaffold-160 | 240120 | + |
| miR-n102-3 | CTTCTCATGTCCATGCTGATACA | TAGCACATGCTCATGAGAAG | Scaffold-215 | 452322 | + |
| miR-n172-1 | TACTTGGATTGTCGGAAGCAGGTG | TGCTGTGTCTGATGCTAAGGATGTG | Scaffold-214 | 736065 | + |
| miR-n91-1 | CATATTTATCATTGTATTGTA | TGCAATCCATTGAATAAATACGAA | Scaffold-328 | 499574 | - |
| miR-n168 | TGACTGTTTAGGAGGGAAGCGGAGG | TGCTTCCATTTTTAGAAGGGTCGGG | Scaffold-197 | 785267 | - |
| miR-996 | GGCGGGTGTAGCTTTGGTGCACA | TGACTAGAGTTACACTCGTCA | Scaffold-31 | 544939 | + |
| miR-279b-2 | GACGGGTAGGGTTATAGTGCACG | TGACTAGATCCTTACTCGTCTG | Scaffold-612 | 106717 | - |
| miR-n100-4 | AGTTGTTGAGAGACCAAAATTGTG | TAATTTGGTCTACTCAACAGCACA | Scaffold-24 | 133385 | + |
| miR-971-1 | CACTCTAAGCTCGAACACCAAGC | TTGGTGTTCTACCTTACAGTGA | Scaffold-166 | 893921 | - |
| miR-n198a | TGAGGTCATGCAGAGCTGAATT | ATTGGTTCCTCATGGCCTCATGAT | Scaffold-1096 | 38899 | + |
| miR-n137c | TGGGGCCATGAGGGACTGAATTC | TTAGTTCCTTATGGCCTCATGA | Scaffold-1096 | 37115 | + |
| miR-n137e | TGGGGTCATGAGGGACTGAATT | TTTGTTCCTTCATGGCCTCACG | Scaffold-215 | 556085 | + |
| miR-n54 | ATAGGGACTTTCTGATGATAAAT | TTTGTCTCAGAAAAGTCCTTGT | Scaffold-20 | 9120798 | - |
| miR-n131-2 | CAATATCAGCCGGTGATTGCGA | GAGATTACCTGCTGATATTTAGG | Scaffold-57 | 24646 | - |
| miR-n48 | CAGGTACTGTTTTTGATATTGTC | TAATACCATTAACAGTACCGTT | Scaffold-160 | 173414 | + |
| miR-n188a | TGGGATCATGCAGAGCTGGATT | TCACAGCCTCCATAATCCCAAG | Scaffold-215 | 412452 | + |
| miR-n126-2 | CAGGAAGCGGGGGTGGAAATGA | ACTTCGACCTCGTCTTCCTCGA | Scaffold-215 | 766189 | - |
| miR-n34-2 | TGCAACTCACCAGGGGTTTGTT | CAAATCTCTGGGGGAGTTACAATT | Scaffold-215 | 482128 | + |
| miR-n90b-4 | TGTATTCACCTTTTAGCACGTTCA | TGTGATGTGAGGTGAGTGCAAT | Scaffold-215 | 572691 | + |

| | | | | | |
|---|---|---|---|---|---|
| miR-133 | AGCTGGTTGAACCCGGGCCAAA | TTGGTCCCCTTCAACCAGCTGT | Scaffold-112 | 1456076 | - |
| miR-n21 | TGGCAGAAACGCTTGATGTGGAGGC | TCCTCATCTATTTGCTCTCTACCT | Scaffold-811 | 16986 | - |
| miR-n63b-4 | TGTTGTTGGGAGACATGAAATG | TTTCATGTCTATGCCCAACAGCA | Scaffold-542 | 175364 | - |
| miR-n63b-1 | TGTTGTTGGGAGACATGAAATG | TTTCATGTCTATGCCCAACAGCA | Scaffold-15 | 7251021 | + |
| miR-13b-1 | ACGTCAAAATGGTTGTGAAATG | TATCACAGCCATTTTTGACGTACC | Scaffold-287 | 304281 | - |
| miR-n153 | ATGGTGCTACCGAGAATGACGGCCT | TTCACGGTGGTATCATTTTGG | Scaffold-664 | 98324 | + |
| miR-n38d | TGTATCAACCGACCTGTAAA | TTTAAAGGACAGTTGATACAAT | Scaffold-1130 | 9955 | + |
| miR-n173a | GAAGTCATCTCAGTATTATATG | AGTGATGCTGTGGTGACTTCA | Scaffold-24 | 125534 | + |
| miR-n77b | TACTGAAAGTAGAAGTCAAATG | ATTTGCTTTCTTCTTTCTTGCT | Scaffold-7 | 1439021 | - |
| miR-n14 | TCTTCTTTGCTTGGACAGACT | TGACCAAGCAAAGTAGACCCT | Scaffold-949 | 7357 | + |
| miR-n115a-2 | TGTATGAAGAAGGTGTGAGTGATA | CACTTACATCTTCTTCATCTGA | Scaffold-215 | 443696 | + |
| miR-n74-3 | TCTGAGACTCTTGTGGTTCATG | TGGAACACATTGGTCTCAGTGT | Scaffold-24 | 95978 | + |
| miR-n102-1 | CTTCTCATGTCCATGCTGATACA | TAGCACATGCTCATGAGAAG | Scaffold-215 | 475150 | + |
| miR-n195a-1 | AGCTGGCATACTTGTTTCCTATG | TAGGAACAATTATGCCGGTTTT | Scaffold-15 | 7241825 | + |
| miR-210-2 | CTGCTGGACACTGCACAAGA | TTGTGCGTGTGACAGCGGCT | Scaffold-411 | 199928 | + |
| miR-n159 | TTGGGCGGTGGGGAATCGTC | CGAGTCTTCTCCGTCCGAC | Scaffold-67 | 4923705 | + |
| miR-n189c-4 | TTGAACTTCATTCTCATTTCGTTTG | TGAAATGATAATGAAACTCAAGG | Scaffold-215 | 710922 | - |
| miR-n38f | TGTATCAACCGACCTGTAAATTCA | TTTAAAGGACAGTTGATACAAT | Scaffold-896 | 12302 | - |
| miR-n166 | AGTAAACAGGAGATCACGAGTTC | TCTCGTCGGGGCCTCATTTTTT | Scaffold-19 | 822524 | + |
| miR-n197 | TAAGACTTCAGATTTCTGCTGA | AGTATTAACCTGTAGTTCTCTTT | Scaffold-430 | 360292 | - |
| miR-n115b | TGTATGAAGAAGGTGTGAGTCATA | CACTTACATCTTCATCATCTGA | Scaffold-215 | 532760 | + |
| miR-13a-2 | CGTCAAATTGGTTGTGAGTTATG | TATCACAGCCACTTTGATGAAC | Scaffold-402 | 386237 | - |
| miR-750 | AGTTGGAAGCGGGATCTAGGC | CCAGATCTAACTCTTCCAGCT | Scaffold-29 | 2431099 | + |
| miR-n107d-2 | TGAGGTCTCGCAGAACTGAATT | TTCAGTCTGCAAAACCTCAAGA | Scaffold-215 | 679903 | - |
| miR-n123 | GCCAAGTACCATCCTAGTGAC | TGACTAGTATGTTACTTGGT | Scaffold-328 | 225559 | + |

| | | | | | |
|---|---|---|---|---|---|
| miR-n85 | TATCTTCGTAACGATATATCGCTAG | CTTCCGATATATCGTTCTGACA | Scaffold-17 | 2570754 | + |
| miR-n98a-4 | AGGAAAGAGAAAGAGGGCAAAT | TTGATCATTTTCTCTTTTCTGT | Scaffold-1740 | 13325 | + |
| miR-n67-3 | AGTAATTATCGCGTCAATCTCA | AGATTGTGTGATAGTTACCATTCA | Scaffold-215 | 694733 | - |
| miR-n195a-2 | AGCTGGCATACTTGTTTCCTATG | TAGGAACAATTATGCCGGTTTT | Scaffold-15 | 7257218 | + |
| miR-1175 | | TGAGATTCACTCCTCCTACTTACT | Scaffold-29 | 2436280 | + |
| miR-n38a-1 | TGTATCAACCGACCTGTAGATT | TTTAAAGGACAGTTGATACA | Scaffold-215 | 492491 | + |
| miR-n196a-7 | CGAGGAACATTTTCTGGTCAT | TGACTAGATACTGTTCCTCAGA | Scaffold-215 | 729880 | - |
| miR-279a-1 | GTTGGGTGGGGGTCTAGTGTACA | TGACTAGATCCACACTCAACA | Scaffold-133 | 477023 | + |
| miR-283b-1 | CAATATCAGCCGGTAATCGCGA | GAGATTGCCCTCTGATATTTAGG | Scaffold-57 | 36360 | - |
| miR-n139 | AGAGTGTTTCAGGAGGAATCTGCCT | AGCGGATTCTTCCTGAAACACT | Scaffold-27 | 1837510 | + |
| miR-n105b-1 | CTTCTCATGATCCCGGTGATAC | TACACTGGGATTATGAGATTTA | Scaffold-160 | 275765 | + |
| miR-2765a-2 | TGGTAACTCCGAACCACCGTTGGC | CAGCGGTGGTGGAGCTACCCTT | Scaffold-76 | 856515 | - |
| miR-n121b-2 | ATTGGTTAATTGAGCTTTAGCG | TTAAAGCTTAGTTACCGATCA | Scaffold-531 | 85659 | + |
| miR-n124 | CTTGCGCCAAGATCATATAGATAG | TACGATTTTCTTGGAGCAATTT | Scaffold-71 | 498081 | + |
| miR-n194j | TGGTATGGTGGGTTTTTCTCAAT | TGAGGAGTTCCCCCCATTCATA | Scaffold-542 | 205490 | - |
| miR-n45 | GCGCTGAAGTATGAGCCCCGTGGA | CCACGAGGCTCATACCAGGCGCT | Scaffold-4843 | 1207 | + |
| miR-n196a-2 | CGAGGAACATTTTCTGGTCAT | TGACTAGATACTGTTCCTCAGA | Scaffold-215 | 413841 | + |
| miR-n89 | CTTCTCATCCCTCACAAGAACAGGA | TTTTTGTGAAGGTTGAGGAGGATA | Scaffold-215 | 539622 | + |
| miR-71-2 | TGAAAGACATGGGTAGTGAGATG | TCTCACTACCTTGTCTTTCATG | Scaffold-402 | 388264 | - |
| miR-n38b-1 | TGTATCAACCGACCTGTAGATT | TTTAGAGGGCAGTTGATACAAT | Scaffold-215 | 415892 | + |
| miR-n36-2 | TAGGTGCTGTTTTTGATATTGTC | TAATATGAAAAATAGCCCTGCTTT | Scaffold-160 | 248366 | + |
| miR-29 | ACTGGTTTCAAAATGGTGGATAGA | TAGCACCATTTGAAATTAGTG | Scaffold-4 | 297947 | + |
| miR-n34-1 | TGCAACTCACCAGGGGTTTGTT | CAAATCTCTGGGGGAGTTACAATT | Scaffold-215 | 687416 | - |
| miR-n43c | TGAGATCATCCTGTTGATCACT | TAATCACATGATTGATCTCAA | Scaffold-160 | 182192 | + |
| miR-n145 | TAGTTGCTGGGAAGGCCTTAGTGG | TGGGCTGGTTCAGATGCTGGAAAT | Scaffold-54 | 1907292 | + |

| | | | | | |
|---|---|---|---|---|---|
| miR-n15 | AGTTTGAGAGCGGTCTACAAGATC | TTGCAGACCGCTCCCGACTGTCC | Scaffold-345 | 201913 | - |
| miR-n90b-7 | TGTATTCACCTTTTAGCACGTTCA | TGTGATGTGAGGTGAGTGCAAT | Scaffold-215 | 679676 | - |
| miR-n26 | GTAAATGATGATTTTGTGTCATG | GCTGATAGTGACTATCTGAAACC | Scaffold-332 | 24866 | + |
| miR-n78a-3 | AGTTGGACAGTTCAAGATTGTTG | CCAACCTTGGCTTTCCGACTGTAG | Scaffold-1740 | 5777 | + |
| miR-n200 | TGAGGTTTTGTAGAACTGAATTTCA | TTCAGTCTGCAAAATCTCAAGA | Scaffold-215 | 462057 | + |
| miR-n95-2 | ACTGAGGCTCTTGCGGTGAGATG | TCTACTACATTAGCCTCAGTGT | Scaffold-71 | 139504 | + |
| miR-n115a-1 | TGTATGAAGAAGGTGTGAGTGATA | CACTTACATCTTCTTCATCTGA | Scaffold-215 | 435268 | + |
| miR-n129 | TGAGGAACATTGTTTGGTCATT | TGACTAGCCTTTGTTCCTTATA | Scaffold-215 | 736190 | - |
| miR-279b-1 | GACGGGTAGGGTTATAGTGCACG | TGACTAGATCCTTACTCGTCTG | Scaffold-133 | 478787 | + |
| miR-n126c | CAGGAAGTGGGGGTGGAAATGA | CTTCCACCTCCTCTTCCTCGAA | Scaffold-215 | 501263 | + |
| miR-n204b | TGGGGTCATGAGGGAATGAATTTA | TTTGTTCCTTATGGCCTCATGA | Scaffold-215 | 690487 | - |
| miR-n194a | TGGTATGGCGGGTTCTTCTCAAT | TGAGGAGTTCCCCCCATTCATA | Scaffold-15 | 7235198 | + |
| miR-263b | CTTGGCACTTGAAGAATTCACAGA | TGGGTCTTCTGGTGCCAAAGT | Scaffold-15 | 3245463 | - |
| miR-n130-1 | TGAGGAACACTGACTAGTCATT | TGACTAGTATTTGTTCCTCAT | Scaffold-215 | 410529 | + |
| miR-n172-2 | TACTTGGATTGTCGGAAGCAGGTG | TGCTGTGTCTGATGCTAAGGATGTG | Scaffold-491 | 128882 | - |
| miR-33 | TTGCATTGTAATTGCATTG | CAATTCATTTGCAATGCACAGTC | Scaffold-1 | 2409685 | - |
| miR-n185-1 | CGTGGCGCCTCAGGTATTAATT | TTAATATCTGTGGGGCTGCAAT | Scaffold-215 | 423105 | + |
| miR-n183-7 | CGGTCTGTCAAGAGTATTTCTATG | TAGATTTCTCTTGACAGGCTTGA | Scaffold-71 | 143842 | + |
| miR-n51 | CAGGTACCGTGTTTGATATTGTC | TAATATCATTCACGGTACTGAA | Scaffold-160 | 230573 | + |
| miR-n175-1 | AGCACTTTCAGCTGGCTTGTTACA | TAAAGCTAGATGAAGGTGTTAA | Scaffold-3004 | 2940 | + |
| miR-n127-4 | TGAGGCCATGCAGAGCTGAAT | TTTGGTTCCTCATGGCCTCATGAT | Scaffold-1130 | 9712 | + |
| miR-n152 | TGAGAAGACACGCCGTTACA | TGTGACAGACTGTCTTCTCAGC | Scaffold-132 | 1329887 | + |
| miR-n88 | GCTTGTATTCTTGTACTTGGTGGAA | TAAGGTACATGAATACAAGAGT | Scaffold-205 | 727309 | - |
| miR-n209-1 | CCCCTCAGCTCTCCCTGCTGCTGCC | TGCTGCAGAGAGCGCTGAGCCG | Scaffold-12 | 320510 | - |
| miR-n192a-1 | TGGCAATGCAGGAGCTTTAATTG | ATGAAGCTTTTGCACTGTCAGA | Scaffold-1436 | 3478 | + |

| | | | | | |
|---|---|---|---|---|---|
| miR-315 | TTTTGATTGTTGCTCAGAAAGCCG | GCTTTCGAGCAATAATCTCAATC | Scaffold-162 | 41663 | + |
| miR-n23 | GTTTATGATGATCTGGTACC | TACCTATTATTACTGAAA | Scaffold-332 | 24486 | + |
| miR-n202a-3 | TTTAAAGGGGCCTCACAGTAACG | TTACTGTGCGGCTACTTTATCT | Scaffold-215 | 522270 | + |
| miR-n62 | TGGGAGTGAGGGGAACTCCTTCATT | TGGAGGATTGAACCTTTTCCACT | Scaffold-976 | 54089 | - |
| miR-317-1 | AGGGAACCACCCTGGGTTCACT | TGAACACAGCTGGTGGTATCT | Scaffold-317 | 212219 | - |
| miR-n140-2 | TTTGGAGGGGACTTATTGCAT | TGCAATATTCCTTTTCCGATA | Scaffold-461 | 305062 | - |
| miR-277-1 | | TAAATGCACTATCTGGTACGACA | Scaffold-317 | 193570 | - |
| miR-n196b | CGAGGAACATTTTCTGGTCAT | CGACTAGATACTGTTCCTCAGAT | Scaffold-215 | 448123 | + |
| miR-190 | AGATATGTTTGATATTCTTGGTTG | ACCGAGGATCAAACATATTATAA | Scaffold-27 | 2037308 | + |
| miR-n118 | CTTGGGAGTGATGTGTTGTATG | TATTACACACCTCTTCCAAGAGA | Scaffold-205 | 711571 | - |
| miR-n195a-9 | AGCTGGCATACTTGTTTCCTATG | TAGGAACAATTATGCCGGTTTT | Scaffold-542 | 207825 | - |
| miR-3049-1 | TCGGGAAGGCAGTTGCGGCGGATT | TCCGTCCAACTCCTTTCCGTCT | Scaffold-194 | 314739 | - |
| miR-n93 | AAGGAAAGTCCCGTGCTAAGT | TTTGCTCCGTTTACTTTCCTACA | Scaffold-690 | 128399 | + |
| miR-n107a-2 | TGAGGTCTCGCAGTACTAAATT | TTTAGTCTGCAAAACCTCAAGATA | Scaffold-215 | 599385 | - |
| miR-n193 | AGCTGGCATGACTGTTATCCATGA | TGGATGGAGTTATGCCAGTTCT | Scaffold-15 | 7233943 | + |
| miR-252b-2 | TAAGTAGTAGTGCCGTAGCGA | | Scaffold-718 | 139271 | - |
| miR-n151-1 | AGCCTAATACCCGCCAAAGATA | TCTTTGGTGTGTATTGGGCTGT | Scaffold-365 | 461596 | - |
| miR-n192b-2 | TGGCAATGCAGGAGCTTTAATTG | ATAAAGCTTTTGTACTGTCAGA | Scaffold-1436 | 8887 | + |
| miR-n125-1 | CTTGGGAGAGTTTTATTGTATG | TACGTAAAGCCCCTCCCAAGGA | Scaffold-828 | 65887 | - |
| miR-124 | CGTGTTCACTGTTGGCCTTTATGC | TAAGGCACGCGGTGAATGCC | Scaffold-144 | 368408 | + |
| miR-n183-5 | CGGTCTGTCAAGAGTATTTCTATG | TAGATTTCTCTTGACAGGCTTGA | Scaffold-1436 | 12602 | + |
| miR-n163 | CCACTGATTCAGTACGAAGTACT | TTCCTACTGAATCCGTCTGAA | Scaffold-124 | 806648 | + |
| miR-927-1 | TTTAGAATTCCTACGCTTTACC | | Scaffold-2110 | 209827 | + |
| miR-n90b-1 | TGTATTCACCTTTTAGCACGTTCA | TGTGATGTGAGGTGAGTGCAAT | Scaffold-215 | 487486 | + |
| miR-n136b | ATCATGAGGGGATTCGACGTTAACA | TTTGTTGGCCCCTCATAATGG | Scaffold-1164 | 17081 | - |

| | | | | | |
|---|---|---|---|---|---|
| miR-n80-2 | AGCGCTTGTTGATTGTGACC | TTGGTCCCACCCACACGTGCAG | Scaffold-15 | 1201204 | + |
| miR-279c | ATGTGAGGATAATTGATAAA | TAACTAGATCCACACTCAC | Scaffold-56 | 1731451 | + |
| miR-9b-1 | TCTTTGGTGATGTAAGCTGTATGA | ATAAAGCTACATTACCGAAGTC | Scaffold-1084 | 9905 | + |
| miR-n135 | TGAAGTGCTTTCTAGTACGGGCA | CCCGTACTTTAAAGCACTTCACC | Scaffold-139 | 141164 | - |
| miR-n137a | TGGGGCCATGAGGGACTGAATTT | TTTGTTCCTTTATGGCCTCATGA | Scaffold-206 | 140796 | - |
| miR-n107d-3 | TGAGGTCTCGCAGAACTGAATT | TTCAGTCTGCAAAACCTCAAGA | Scaffold-215 | 734648 | - |
| miR-n98c | AGGAAAGAGAAAGAGGGTCAATG | TTGATCACTTTCTCTTTTCTGC | Scaffold-1740 | 7993 | + |
| miR-n99-3 | AGCTGGTATAATTGTTGTCCATGA | TGACTGCAATAATACCAGCTACA | Scaffold-24 | 127475 | + |
| miR-n162 | ACACATCATGTCCGTGGACTGT | AGCACAATCCACGGACATGATG | Scaffold-53 | 614745 | + |
| miR-n46 | AATCTGCTTGTCGCCTCCTAA | TGGAGTGAATCAAGGAGATACAAAT | Scaffold-147 | 1128659 | - |
| miR-n6 | TTTCCTGACTTGTTTTTTCTCTTT | AAGTAACAAGTCACGAAAGAC | Scaffold-72 | 1812856 | + |
| miR-n35-2 | TGAGATCGGTTTCAACTAGGAAATG | TCGTTTGATTTGATTGAAGGAGTC | Scaffold-43 | 1967163 | + |
| miR-n126d | TAGGAAGCGGGGGTGGAAATG | CTTCCACCTCCTCTTCCTCGAA | Scaffold-215 | 755158 | - |
| miR-n99-1 | AGCTGGTATAATTGTTGTCCATGA | TGACTGCAATAATACCAGCTACA | Scaffold-828 | 58116 | - |
| miR-n90b-6 | TGTATTCACCTTTTAGCACGTTCA | TGTGATGTGAGGTGAGTGCAAT | Scaffold-215 | 653256 | - |
| miR-n105a | CTTCTCATGATCCCAGTGATACAT | TACACTGGGATTATAAGATTTA | Scaffold-160 | 256196 | + |
| miR-n107a-4 | TGAGGTCTCGCAGTACTGAATT | TTTAGTCTGCAAAACCTCAAGATA | Scaffold-215 | 744347 | - |
| miR-n35-1 | TGAGATCGGTTTCAACTAGGAAATG | TCGTTTGATTTGATTGAAGGAGTC | Scaffold-26 | 2366261 | - |
| miR-279a-2 | GTTGGGTGGGGGTCTAGTGTACA | TGACTAGATCCACACTCAACA | Scaffold-612 | 108494 | - |
| miR-n8 | TGGGTTCTCAAATGTAGTATC | GACTACAAAGAGGTTGTCCCACA | Scaffold-47 | 992408 | - |
| miR-n38e | TGTATCAACCGACCTGTAGATTCA | TTCAGAGGGCAGTTGATACAAT | Scaffold-215 | 768820 | - |
| miR-n113-1 | ACTGGTTTAATTGAGCTTTGACA | TTAAAGCTCCCTTAACACCGGTCA | Scaffold-531 | 82954 | + |
| miR-184a | CCTTATCATTCTCTCGGCCAGTT | TGGACGGAGAACTGATAAGGGC | Scaffold-2 | 698491 | + |
| miR-n186 | CATCAGTACCTTGCCGGCTTTTG | TAGGCGGGGATGGTACTGTTTT | Scaffold-24 | 3665084 | + |
| let-7 | TGAGGTAGTAGGTTGTATAGTA | CTGTACAGCCTGCTAACTTTCC | Scaffold-15 | 5604710 | + |

| | | | | | |
|---|---|---|---|---|---|
| miR-n204-2 | TGGGGTCATGAGGGAATGAATTTA | TTTGGTTCCTTATGGCCTCACGA | Scaffold-215 | 702962 | - |
| miR-n36-1 | TAGGTGCTGTTTTTGATATTGTC | TAATATGAAAAATATCCCTGCTTT | Scaffold-160 | 220852 | + |
| miR-n136a-2 | ATCATGAGGGGATTCGACGTTAACA | TTTGTTGGCCCCTCATAATGG | Scaffold-103 | 1601108 | + |
| miR-306 | ACAGGTACTGAGTGACTCTGAG | CAGGGGCGCTAAGTACCTTCCA | Scaffold-113 | 385915 | + |
| miR-n122 | TCGTGATCTATCCTTGACTTGTT | CAGTCTCCGATAAATCATGAAT | Scaffold-325 | 77876 | + |
| miR-n142-2 | AATCTCATAAGGTAATGATATA | TATCATTATCGAATGGGGTTTCA | Scaffold-57 | 22265 | - |
| miR-n175-2 | AGCACTTTCAGCTGGCTTGTTACA | TAAAGCTAGATGAAGGTGTTAA | Scaffold-531 | 88246 | + |
| miR-n127-2 | TGAGGTCATGCCAAGCTGAAT | TTTGGTTCCTCATGGCCTCATGAT | Scaffold-215 | 492248 | + |
| miR-n117 | AGCTGGGTAGATACGCCGTCAT | TATGACGACGTTTCTTCCCAGAA | Scaffold-171 | 605931 | + |
| miR-n179 | AATGCTATACTCACGAATACT | TATTCGTGGTTATATTCATTTT | Scaffold-703 | 74590 | - |
| miR-n194i | TGGTATGGCGTGTTCTTCTCA | TGAGGAGTTCCCCCCATTCAT | Scaffold-542 | 205177 | - |
| miR-n50 | TAGGTACTGTTTTTGATATTGTC | TAATATCAAAAATAGTCCTGCT | Scaffold-160 | 211858 | + |
| miR-n132-2 | ATTGGATAATAGAGCTTTGACG | TTAAAGCTTTATTACCGATTA | Scaffold-622 | 139080 | + |
| miR-n44 | TGGCCAATCACAACTCTTGTT | ACAAGAGCTATGATTGGCCACA | Scaffold-314 | 324850 | - |
| miR-n90b-8 | TGTATTCACCTTTTAGCACGTTCA | TGTGATGTGAGGTGAGTGCAAT | Scaffold-215 | 730721 | - |
| miR-252a-2 | CTAAGTACTGGTGCCGTTAGGAG | CCTGCAGCTCGAGTGCTTATCA | Scaffold-718 | 123786 | - |
| miR-n195a-6 | AGCTGGCATACTTGTTTCCTATG | TAGGAACAATTATGCCGGTTTT | Scaffold-542 | 175247 | - |
| miR-n203-1 | TGGTGGAGACGGGAAAAATTATA | TATTTTTCTCGTTTGCGCCAGA | Scaffold-15 | 7235043 | + |
| miR-n198b-3 | TGAGGTCATGCAGAACTAAATT | TTTGGTTCCTCATGGCCTCACGA | Scaffold-215 | 711112 | - |
| miR-n190 | TGGGATCATGCAGAGCTGATTT | AATAGTCTGTATGGTTCCAAGA | Scaffold-215 | 663165 | - |
| miR-9b-2 | TCTTTGGTGATGTAAGCTGTATGA | ATAAAGCTACATTACCGAAGTC | Scaffold-113 | 388129 | + |
| miR-n107b | TGAGGTCTCGCAGAACTGAATT | TTTAGTCTGCAAAACCTCTAGA | Scaffold-215 | 686021 | - |
| miR-307 | ACTCACTCAATCCGGGTGTG | CACAACCTCCTTGAGTGAGCGA | Scaffold-259 | 380525 | + |
| miR-n24 | TGCTTCAAGGAAATGGTTGCTGGTC | TCGGTATCAGCCATCTCGAAGGAGG | Scaffold-1 | 3963739 | - |
| miR-n84-1 | TGGCAATTTTCACATCAATCTCA | CGATTGTGTGATTGTTGCCATT | Scaffold-160 | 267833 | + |

| | | | | | |
|---|---|---|---|---|---|
| miR-n33 | TATGATGATTCAAATTATAGGAATT | AATGACGATAATTTATCTCTGATA | Scaffold-244 | 262000 | - |
| miR-n73a | GCTGGGACTCTTGGGCTTTATG | TAAAGCTTATTAGCCCCGGTGT | Scaffold-728 | 15055 | - |
| miR-n83 | TGAGGTTATGTTGGAGTTATT | ATACCTCCTTTGTAGCCTCATGA | Scaffold-160 | 267367 | + |
| miR-n196a-8 | CGAGGAACATTTTCTGGTCAT | TGACTAGATACTGTTCCTCAGA | Scaffold-215 | 770734 | - |
| miR-n134 | CAATATCAGCCGGTAATTGTGAT | ACAAGTGCTCGTTGATATCTGGA | Scaffold-57 | 40167 | - |
| miR-263a-1 | AATGGCACTGGAAGAATTCACGGG | CGTGGTCCTCTGGTGTCATACC | Scaffold-15 | 3276507 | - |
| miR-n35-3 | TGAGATCGGTTTCAACTAGGAAATG | TCGTTTGATTTGATTGAAGGAGTC | Scaffold-26 | 2359903 | + |
| miR-n121 | ATTGGTTAATTAAGCTTTAGCG | TTAAAGCTTAGTTACCGATC | Scaffold-3004 | 36 | + |
| miR-n59-1 | TGATGGTAATTGCCTGGTCGATGT | TCACCGGGCACTTACTACCAACT | Scaffold-3004 | 2480 | + |
| miR-277-2 | | TAAATGCACTATCTGGTACGACA | Scaffold-527 | 130123 | - |
| miR-n192b-3 | TGGCAATGCAGGAGCTTTAATTG | ATAAAGCTTTTGTACTGTCAGA | Scaffold-1436 | 12425 | + |
| miR-n202a-2 | TTTAAAGGGGCCTCACAGTAACG | TTACTGTGCGGCTACTTTATCT | Scaffold-215 | 414172 | + |
| miR-n195b | AGCTGGCATACTTGTTTCCTATG | TAGGAACAATTATGCCGGTTTC | Scaffold-15 | 7285620 | + |
| miR-n98b | AGGAAAGAGAAAGAGGGTGAA | TCAATCTTTTTCTCCTTTCTG | Scaffold-7 | 1451685 | - |
| miR-n32a-1 | GAAGTCATCTCAGCATTATATGC | AAAGATGCTGTTATGACTTCACT | Scaffold-828 | 63377 | - |
| miR-n96 | AGACTGAACAGAGGACTCTTCC | ATGAAGAGTTCTCTGTTCACT | Scaffold-72 | 11033801 | + |
| miR-n189b | TTGAGCTTCATTGCCATTTCGTTTC | TGATATGATAATGAAACTCAAGG | Scaffold-215 | 549006 | + |
| miR-278 | ACGGACAAAGGCTCCACATGGCC | TCGGTGGGACTTTCGTCCGTCT | Scaffold-91 | 460559 | - |
| miR-n43d | TGAGATCATCCTGTTGATTACT | TAATCTCATGATTGATCTCAAGC | Scaffold-160 | 186745 | + |
| miR-n126e | CCAGGAAGCGGGGGTGGAAATC | CTTCCACCTCCTCTTCCTCGAA | Scaffold-35 | 1522325 | - |
| miR-n56b-2 | TGGTAACTTCCACCACTATCT | AGATAGGGTGGTTGTTACCATTC | Scaffold-215 | 746718 | - |
| miR-n183-6 | CGGTCTGTCAAGAGTATTTCTATG | TAGATTTCTCTTGACAGGCTTGA | Scaffold-71 | 140308 | + |
| miR-n191 | AGACCAAGAGCCAGAACAGAAC | TCTGGTCGTGTTCTTGGTCACA | Scaffold-72 | 6663345 | - |
| miR-n37-4 | TGTACCAGCCGTCCTGGTGGATT | TCCCCAGTGCAGTTGATACA | Scaffold-215 | 642709 | - |
| miR-n64 | GATGAATGCTGTGATGAGTTTGC | CATACCCACAATAGCATTCACT | Scaffold-157 | 808392 | + |

| | | | | | |
|---|---|---|---|---|---|
| miR-993 | | GAAGCTCGTCTCTACAGGTATCT | Scaffold-72 | 7053267 | + |
| miR-283b-2 | CAATATCAGCCGGTAATCGCGA | GAGATTGCCCTCTGATATTTAGG | Scaffold-57 | 38311 | - |
| miR-n58 | TGTGATGTGTATGTGGGCTTACT | TGGCCCACTATCACAGCACTTGA | Scaffold-29 | 1947602 | - |
| miR-n183-2 | CGGTCTGTCAAGAGTATTTCTATG | TAGATTTCTCTTGACAGGCTTGA | Scaffold-1436 | 3655 | + |
| miR-137 | ACGCGTATTCTTGGGGAATTAACA | TATTGCTTGAGAATACACGTAGT | Scaffold-385 | 78672 | - |
| miR-n43e | TGAGATCATCATGTTTATTACTTTG | TAATGTCATGACTGATCTCAAG | Scaffold-160 | 230899 | + |
| miR-2a-1 | CTCACAAAGTGGTTGTCATATG | TATCACAGCCAGCTTTGATGAGCGA | Scaffold-287 | 304134 | - |
| miR-971-2 | CACTCTAAGCTCGAACACCAAGC | TTGGTGTTCTACCTTACAGTGA | Scaffold-261 | 360342 | - |
| miR-92a | AGTCCGTGATGAGTGCCAATACT | TATTGCACTTGTCCCGGCCTAT | Scaffold-36 | 255389 | - |
| miR-n115a-3 | TGTATGAAGAAGGTGTGAGTGATA | ACTTACATCTTCTTCATCTGAAT | Scaffold-215 | 695197 | - |
| miR-n94-1 | CTAAGTAGTGATGAAGTGGCTGCT | CACCTACGCATTCCTGCTTAA | Scaffold-718 | 137677 | - |
| miR-n209-2 | CCCCTCAGCTCTCCCTGCTGCTGCC | TGCTGCAGAGAGCGCTGAGCCG | Scaffold-33 | 2098813 | - |
| miR-n192c-1 | TGGCAATGCAGGAGCTTTAATTG | ATAAAGCTTTTGCACTGTCAGA | Scaffold-1436 | 6561 | + |
| miR-n59-3 | TGATGGTAATTGCCTGGTCGATGT | TCACCGGGCACTTACTACCAACT | Scaffold-622 | 141927 | + |
| miR-n81c | TGATGGAAGGTAGTTCCTCAAT | TGGGGAGCTCCTCTTCCATCAGA | Scaffold-542 | 205017 | - |
| miR-n108 | ATCTGGTCTGATATTTGCCAAGA | TTGGCAAAGTCGGAGTCAGCT | Scaffold-24 | 3665350 | + |
| miR-71-1 | TGAAAGACATGGGTAGTGAGATG | TCTCACTACCTTGTCTTTCATG | Scaffold-287 | 308767 | - |
| miR-n183-3 | CGGTCTGTCAAGAGTATTTCTATG | TAGATTTCTCTTGACAGGCTTGA | Scaffold-1436 | 5196 | + |
| miR-n180c | TTGTATTCATCTTTCAGCACATT | TGTTATGTTAGATGAGTAC | Scaffold-215 | 714540 | - |
| miR-n107c-3 | CGAGGTCTCGCAGAACTGAAT | TTCAGTCTGCAAAACCTCTAGA | Scaffold-215 | 612697 | - |
| miR-n38a-2 | TGTATCAACCGACCTGTAGATT | TTTAAAGGACAGTTGATACA | Scaffold-215 | 620916 | - |
| miR-n203-2 | TGGTGGAGACGGGAAAAATTATA | TATTTTTCTCGTTTGCGCCAGA | Scaffold-15 | 7243479 | + |
| miR-n113-2 | ACTGGTTTAATTGAGCTTTGACA | TTAAAGCTCCCTTAACACCGGTCA | Scaffold-622 | 137519 | + |
| miR-n7 | TTGTACTATCGATTGAATTGCGGGC | TGCGCTGTCGGTCGAGTTGACGTGT | Scaffold-239 | 162419 | - |
| miR-n57-1 | CGAGATCATAATGAAAGTTACGCT | TGGGCTTCCAGTATGATCTGTC | Scaffold-215 | 577857 | + |

| | | | | | |
|---|---|---|---|---|---|
| miR-n35-4 | TGAGATCGGTTTCAACTAGGAAATG | TCGTTTGATTTGATTGAAGGAGTC | Scaffold-139 | 980503 | - |
| miR-n189c-1 | TTGAACTTCATTCTCATTTCGTTTG | TGAAATGATAATGAAACTCAAGG | Scaffold-215 | 660813 | - |
| miR-n175-3 | AGCACTTTCAGCTGGCTTGTTACA | TAAAGCTAGATGAAGGTGTTAA | Scaffold-622 | 142382 | + |
| miR-n75-1 | GCTGGGACTCTTGGGCCTTATG | TAAGGTACATTAGTCTCAGTGT | Scaffold-728 | 15414 | - |
| miR-n102-2 | CTTCTCATGTCCATGCTGATACA | TAGCACATGCTCATGAGAAG | Scaffold-215 | 508525 | + |
| miR-n41 | AGCTGAGAAGAAACGTCGCCTC | ACCGGAGTCTCTTCTCAGTGTG | Scaffold-256 | 621010 | - |
| miR-n74-2 | TCTGAGACTCTTGTGGTCCATG | TGGAACACATTGGTCTCAGTGT | Scaffold-828 | 96236 | - |
| miR-n206 | TAGATATCTACTTTGTGCTGGG | TCCACCTCATTGTAGACATCT | Scaffold-20 | 4585161 | - |
| miR-n79 | TGACCAAGAACACGACCAGATT | TCTGTTCTGGCTCTTGGTCTGAC | Scaffold-72 | 6663347 | + |
| miR-n194g-1 | TTGTATGGAGGGCTCTTCTCA | TGAGGAGTTCCCCCATATTCAT | Scaffold-542 | 192583 | - |
| miR-n137d-1 | TGGGGTCATGAGGGACTGAATT | TTTGTTCCTTTATGGCCTTATGA | Scaffold-215 | 489925 | + |
| miR-n97 | GGACATAAAGGATGAGATGGAC | CTATTTCATCCTGTATTTCCGCT | Scaffold-142 | 808837 | - |
| miR-n90b-9 | TGTATTCACCTTTTAGCACGTTCA | TGTGATGTGAGGTGAGTGCAAT | Scaffold-215 | 744122 | - |
| miR-n110 | CTGAGAAGAGACGCCGTCACAA | TGAATGGCGTCTCTTCTCGACT | Scaffold-41 | 2346433 | - |
| miR-n19-2 | TCAATACGGAGGATGAGGAAGAGCT | CTGTCATTTTCTCCGTCCTGGAAA | Scaffold-97 | 1695634 | + |
| miR-100 | AACCCGTAGATCCGAACTTGTG | CAAGGCCGTTTCTGAGGGTCTG | Scaffold-15 | 5602440 | + |
| bantam | CCGGGTTTCACAGTGATCTGCCA | TGAGATCATTGTGAAAGCTGAT | Scaffold-147 | 1116726 | + |
| miR-184b | CCTTATCATTCTTCTGTCGGGA | CGGACGGAGAACTGATAAGGGC | Scaffold-52 | 1997691 | - |
| miR-n90b-3 | TGTATTCACCTTTTAGCACGTTCA | TGTGATGTGAGGTGAGTGCAAT | Scaffold-215 | 530703 | + |
| miR-n189a | TTGAACTTCATTCTCATTTC | TGATATGATAATGAAACTCAAGG | Scaffold-215 | 542423 | + |
| miR-n71 | TTGTACTTAGCTCCCAGCACATT | TGTAATGAGAGATAAGTGCATT | Scaffold-43 | 1980004 | + |
| miR-14 | AGGGGAGAGCGGGAGGCTTAGACT | TCAGTCTTTTTCTCTCTCCT | Scaffold-111 | 893818 | + |
| miR-n194c-1 | TAGTATGGCGGGTTCTTCTCA | TGAGGAGTTCCCCCCATTCATA | Scaffold-15 | 7243633 | + |
| miR-n196a-4 | CGAGGAACATTTTCTGGTCAT | TGACTAGATACTGTTCCTCAGA | Scaffold-215 | 586529 | + |
| miR-n63b-6 | TGTTGTTGGGAGACATGAAATG | TTTCATGTCTATGCCCAACAGCA | Scaffold-542 | 207942 | - |

| | | | | | |
|---|---|---|---|---|---|
| miR-n202b | TTTGAAGGGGCCTCACAGTAACG | TTACTGTGCGGCTACTTTATCT | Scaffold-215 | 448467 | + |
| miR-n10 | GTGGGATGATGGTGATGGTATGAC | TTCACCACCATCATCATCCCTTTT | Scaffold-49 | 631600 | + |
| miR-n147 | CTGATATGATGATATTCGGATCCT | ATAACTTGAAATCTGATC | Scaffold-113 | 911698 | + |
| miR-n52a-1 | CAGGTACCGTGTTTGATATTGTC | TAATATCATTCACGGTACTGCT | Scaffold-160 | 257667 | + |
| miR-n195a-7 | AGCTGGCATACTTGTTTCCTATG | TAGGAACAATTATGCCGGTTTT | Scaffold-542 | 182111 | - |
| miR-n3-1 | ATGGAGTCCGAAGTCACAGAACATT | TTCATGTGGCGGAATTGACTTCA | Scaffold-699 | 14472 | + |
| miR-n198b-2 | TGAGGTCATGCAGAACTAAATT | TTTGGTTCCTCATGGCCTCACGA | Scaffold-215 | 661003 | - |
| miR-n29 | TGCAGCTGTAGGCCACAGGATTTAT | CACTTTGGCTAGCACTTCTGAAAAA | Scaffold-455 | 157469 | - |
| miR-n194c-2 | TAGTATGGCGGGTTCTTCTCA | TGAGGAGTTCCCCCCATTCATA | Scaffold-15 | 7253937 | + |
| miR-n12-1 | GAGATTGTTCTATTGGTGATG | TGTGACCAATAGAATGATTTCT | Scaffold-86 | 95953 | + |
| miR-n202a-5 | TTTAAAGGGGCCTCACAGTAACG | TTACTGTGCGGCTACTTTATCT | Scaffold-215 | 586873 | + |
| miR-n194f-2 | TAGTATGGAGGGTTCTTCTCAAT | TGAGGAGTTCCCCCATATTCAT | Scaffold-542 | 192896 | - |
| miR-263a-2 | AATGGCACTGGAAGAATTCACGGG | CGTGGTCTTTCGGTGTCGTACC | Scaffold-329 | 391322 | - |
| miR-n120 | ACAGTCGGTGTTCAGCATCACT | AGTGGTCTGATCACTGCACTGTT | Scaffold-24 | 935917 | - |
| miR-n170 | CCTTGAGCCTTTCGGTCAAGTG | TTTGTTCGTCAGGCTCAAGTCA | Scaffold-50 | 135656 | + |
| miR-n107c-2 | TGAGGTCTCGCAGAACTGAATT | TTCAGTCTGCAAAACCTCTAGA | Scaffold-215 | 547898 | + |
| miR-n11 | TACCACATGATGTTAGATGAAAACT | AATGAAATCAACTTCAAACTGATGG | Scaffold-16 | 1689839 | - |
| miR-n52b-1 | CAGGTACTATGTTTGATATTGTC | TAATATCATTCACAGTACTGCT | Scaffold-160 | 260182 | + |
| miR-n99-4 | AGCTGGTATAATTGTTGTCCATGA | TGACTGCAATAATACCAGCTACA | Scaffold-24 | 130326 | + |
| miR-n17 | TACGAGCCTGGTGACGCCATCTAAA | TGGATGCGTCTCATCTCACCATTTT | Scaffold-365 | 319389 | - |
| miR-n60 | CTGTATCAACCGACCTAACGATT | ATAGTTTGCTCAGTTGATACA | Scaffold-43 | 1976968 | + |
| miR-n69 | GCTGGGACTCTTGTGGGAGAATG | TTCTTCACATTAGTCCCAGTGT | Scaffold-24 | 86615 | + |
| miR-n38b-2 | TTGTATCAACCGACCTTTAGATT | TTTAGAGGGCAGTTGATACAAT | Scaffold-215 | 559136 | + |
| miR-n202a-4 | TTTAAAGGGGCCTCACAGTAACG | TTACTGTGCGGCTACTTTATCT | Scaffold-215 | 568643 | + |
| miR-2765c-1 | TGGTAACTCCACCACCGTTGGC | CAATGGTGCTGAAGTTCCTACA | Scaffold-12 | 851551 | + |

| | | | | | |
|---|---|---|---|---|---|
| miR-n148-2 | CAGGTACTGTGTTTGATATTGTC | TAATATCATTCATAGTACCGCT | Scaffold-160 | 186295 | + |
| miR-193-3 | CGGGACTTGGTGGTATAGTTGG | TACTGGCCTGCTAAGTCCCAAG | Scaffold-759 | 94531 | - |
| miR-n109 | TACAGTTCTTTGCACATTTATG | TAAATGGCGATTGAACTGTGCT | Scaffold-24 | 3668519 | + |
| miR-n74-1 | TCTGAGACTCTTGTGGTCCATG | TGGAACACATTGGTCTCAGTGT | Scaffold-728 | 15244 | - |
| miR-n132-1 | ATTGGATAATAGAGCTTTGACG | TTAAAGCTTTATTACCGATTA | Scaffold-12697 | 671 | + |
| miR-n25 | TTCGCATTCTGTATCTATTGTTCA | GTCACAGACATGGAATGCTAAAA | Scaffold-197 | 854349 | - |
| miR-n148-1 | CAGGTACTGTGTTTGATATTGTC | TAATATCATTCATAGTACCGCT | Scaffold-160 | 182806 | + |
| miR-n104 | TCGGATATCTGAATGGATGGA | TAAATCCGTTCAGATGCCTGA | Scaffold-15 | 3603938 | + |
| miR-9a-3 | TCTTTGGTTATCTAGCTGTATGA | ATAAAGCTAGGTTACCGAAGTTA | Scaffold-143 | 1000550 | + |
| miR-n16 | AAATAAGTCGGACATGTGGAGTTT | ATTCCTTTGGACGAGTGCCTTATCCGA | Scaffold-58 | 2009528 | + |
| miR-n36-3 | TAGGTGCTGTTTTTGATATTGTC | TAATATGAAAAATATCCCTGCTTT | Scaffold-160 | 267997 | + |
| miR-n141-2 | CTTGGAAGGGATTTACTGCATGT | TACAGTCGACCTCTCTCAAGA | Scaffold-461 | 304418 | - |
| miR-n205 | TCACGGATACTAGAATACATTCT | AGAATGTATTCTAGTATCTGCGAT | Scaffold-97 | 86946 | - |
| miR-n12-2 | GAGATTGTTCTATTGGTGATG | TGTGACCAATAGAATGATTTCT | Scaffold-86 | 99885 | - |
| miR-n171 | CTGGTCTGAAAACCTCCTTGCTGT | TTCATCACTAAGGACTTCAGACTGG | Scaffold-55 | 660154 | - |
| miR-n141-1 | CTTGGAAGGGATTTACTGCATGT | TACAGTCGACCTCTCTCAAGA | Scaffold-28 | 1826928 | + |
| miR-n3-2 | ATGGAGTCCGAAGTCACAGAACATT | TTCATGTGGCGGAATTGACTTCA | Scaffold-163 | 56868 | - |
| miR-n150 | TGCCTAGTATTCGTTCTGATCACA | TGCAGAAAGTATACTAGGCCT | Scaffold-3008 | 5773 | + |
| miR-n126b-1 | CAGGAAGCGGGGGTGGAAAT | CTTCCACCTCCTCTTCCTCGAA | Scaffold-1096 | 24527 | + |
| miR-n81b | TGATGGAAGGGAGTTCCTCAAT | TGGGGAGCTCCTCTTCCATCAGA | Scaffold-15 | 7244105 | + |
| miR-n65-1 | TTGGAACTATTGTGTCAATCTC | TCATTGCATGATAGTTACCATTC | Scaffold-16105 | 149 | - |
| miR-n80-1 | AGCGCTTGTTGATTGTGACC | TTGGTCCCACCCACACGTGCAG | Scaffold-15 | 1200658 | + |
| miR-n182 | AGCTGGTCTGACATTTGTCAAGA | TTGACAAGTTCATACTTAGCT | Scaffold-24 | 3667521 | + |
| miR-92b | AGGTCATGACCAGTGCATATTG | AATTGCACTTGTCCCGGCCTGC | Scaffold-36 | 253881 | - |
| miR-n99-2 | AGCTGGTATAATTGTTGTCCATGA | TGACTGCAATAATACCAGCTACA | Scaffold-828 | 61709 | - |

| | | | | | |
|---|---|---|---|---|---|
| miR-n180b | TTGTATTCATCTTTCAGCACATT | TGTTATGTGAGATGAGTACATT | Scaffold-215 | 490286 | + |
| miR-n204a-1 | TGGGGTCATGAGGGAATGAATTTA | TTTGGTTCCTTATGGCCTCACGA | Scaffold-215 | 415649 | + |
| miR-n196a-3 | CGAGGAACATTTTCTGGTCAT | TGACTAGATACTGTTCCTCAGA | Scaffold-215 | 521382 | + |
| miR-n126-1 | CAGGAAGCGGGGGTGGAAATGA | ACTTCGACCTCGTCTTCCTCGA | Scaffold-896 | 7978 | - |
| miR-2a-2 | CTCACAAAGTGGTTGTCATATG | TATCACAGCCAGCTTTGATGAGCGA | Scaffold-402 | 384361 | - |
| miR-316 | TGTCTTTTTCCGCTTTGCTGCCG | ACAGCAAAGTGAAAAAGACCGA | Scaffold-421 | 414222 | - |
| miR-n42 | TGTGAAAGGAGGCGTTAGATTGGCC | GCCAGTAGGAAGATTTTAGGAT | Scaffold-97 | 1697045 | - |
| miR-n78a-2 | AGTTGGACAGTTCAAGATTGTTG | CCAACCTTGGCTTTCCGACTGTAG | Scaffold-7 | 1448908 | - |
| miR-n119 | TAGCGGATTCCGACTTCCATTAAT | ATGGAAGTTGGAATACACTT | Scaffold-24 | 7754743 | + |
| miR-n203-3 | TGGTGGAGACGGGAAAAATTATA | TATTTTTCTCGTTTGCGCCAGA | Scaffold-15 | 7253783 | + |
| miR-n114-1 | CTTGGGAGGGATGTGTTGTATGT | TATTACACACCTCTTCCAAGATA | Scaffold-828 | 67089 | - |
| miR-n19-1 | TCAATACGGAGGATGAGGAAGAGCT | CTGTCATTTTCTCCGTCCTGGAAA | Scaffold-74 | 810059 | - |
| miR-2b-2 | CACATCAAAGTGGCTGTGAAATG | TATCACAGCCAGCTTTGATGAGCGT | Scaffold-402 | 381657 | - |
| miR-n9 | GTGCAATGATGATAAAGAT | TTTTTAACACTGAGCACT | Scaffold-167 | 648171 | + |
| miR-n211 | CCCTGAACTGGCTCTTGCTCTG | GAGCACAGACTTGGTGGGAA | Scaffold-5 | 341538 | - |
| miR-927-2 | TTTAGAATTCCTACGCTTTACC | | Scaffold-593 | 222616 | - |
| miR-n1 | CGGCCTCCGCTATTGTCACGTTC | AACGTGACAATAGCGGAGGCCG | Scaffold-71 | 306889 | + |
| miR-n164 | TTAGCAGAGACTTCCCCTCAGGC | TCTGAGTGGATTTTTTGTAGGTGCT | Scaffold-27 | 2497379 | - |
| miR-n87-2 | GCTGGGACTCTTGTGAGTTATG | TTGCTCACATTAGTCCCAGTGT | Scaffold-205 | 727658 | - |
| miR-n61-2 | GCTGGGACTTTTGAGTAGTTATG | TTCTTCTCATTAGTCCTAGTGT | Scaffold-728 | 24776 | - |
| miR-n94-2 | CTAAGTAGTGATGAAGTGGCTGCT | CACCTACGCATTCCTGCTTAA | Scaffold-225 | 288028 | - |
| miR-125 | TCCCTGAGACCCTAATTTGTGA | CAAATTGGGCAATCAGGCCT | Scaffold-15 | 5626352 | + |
| miR-n183-4 | CGGTCTGTCAAGAGTATTTCTATG | TAGATTTCTCTTGACAGGCTTGA | Scaffold-1436 | 9064 | + |
| miR-n137d-2 | TGGGGTCATGAGGGACTGAATT | TTTGTTCCTTTATGGCCTTATGA | Scaffold-215 | 548819 | + |
| miR-n20 | TTTCTTACTGGTCGGTGTCATCAGC | TTTTCTTGCTCTCCTTTTCC | Scaffold-90 | 731853 | + |

| | | | | | |
|---|---|---|---|---|---|
| miR-n75-2 | GCTGGGACTCTTGGGCCTTATG | TAAGGTACATTAGTCTCAGTGT | Scaffold-828 | 96406 | - |
| miR-n194b-1 | TAGTATGGAGGGCTCTTCTCAAT | TGAGGAGTTCCCCCATATTCAT | Scaffold-15 | 7235510 | + |
| miR-n151-2 | AGCCTAATACCCGCCAAAGATA | TCTTTGGTGTGTATTGGGCTGT | Scaffold-500 | 1281 | - |
| miR-2765b-2 | TGGTAACTTCACCACCGTTGGC | CAACGGTGCTGTAGTTCCTACA | Scaffold-96 | 385524 | + |
| miR-n184 | TGTGTATCTGAAAGCTGCGGTGACC | TGCTGCATAGTTCAGGATGGGCCTA | Scaffold-15 | 939741 | - |
| miR-n146-2 | TGAGTAGACACGCTTTAGCAATA | TGTAAGAGCTTGTCTTCTCAGC | Scaffold-185 | 464090 | + |
| miR-n176 | TTACGTACTGTAATATCAGCC | TTACAGTACGTGGAAACATGC | Scaffold-76 | 857330 | - |
| miR-iab-8 | TTACGTATACTGAAGGTATACCGGA | TCAGGATACATTCAGTATACGT | Scaffold-72 | 8695284 | - |
| miR-n192b-1 | TGGCAATGCAGGAGCTTTAATTG | ATAAAGCTTTTGTACTGTCAGA | Scaffold-1436 | 5019 | + |
| miR-210-1 | CTGCTGGACACTGCACAAGA | TTGTGCGTGTGACAGCGGCT | Scaffold-159 | 125370 | + |
| miR-n128-1 | TGAGGAACATTTTCTGGTCATA | TGACTAGATTCTGTTCCTCAGA | Scaffold-215 | 514562 | + |
| miR-n100-1 | TGTTGTTGGGAGACCAAAAATGT | TAATTTGGTCTACTCAACAGCACA | Scaffold-828 | 58233 | - |
| miR-2c-2 | TCATCAAAGCCGGATTGTCATA | TCACAGCCAGCTTTGATGAGCAC | Scaffold-402 | 388134 | - |
| miR-n177-2 | AAGGAACTACTGGTGTGCATAT | TATCACAGCCGTAATTCCTGTG | Scaffold-622 | 135721 | + |
| miR-n63b-2 | TGTTGTTGGGAGACATGAAATG | TTTCATGTCTATGCCCAACAGCA | Scaffold-15 | 7257098 | + |
| miR-n61-1 | GCTGGGACTTTTGAGTAGTTATG | TTCTTCTCATTAGTCCTAGTGT | Scaffold-728 | 14530 | - |
| miR-9c | TCTTTGGTATGCTAGCTGA | TAAAGCTAGTATACCAAAGTCA | Scaffold-113 | 383457 | + |
| miR-n98a-1 | AGGAAAGAGAAAGAGGGCAAATG | TTGATCATTTTCTCTTTTCTGT | Scaffold-7 | 1444607 | - |
| miR-252a-1 | CTAAGTACTGGTGCCGTTAGGAG | CCTGCAGCTCGAGTGCTTATCA | Scaffold-225 | 274630 | - |
| miR-n87-1 | CCTGGGACTCTTGTGAGTTATG | TTGCTCACATTAGTCCCAGTGT | Scaffold-205 | 717679 | - |
| miR-8 | CATCTTACCGGGCAGCATTAGA | TAATACTGTCAGGTAAAGATGTC | Scaffold-50 | 3010924 | - |
| miR-12-2 | AGAGTATTATATCAGGTACTGGT | | Scaffold-57 | 27640 | - |
| miR-1 | CCGTGCTTCCTTACTTCCCATAG | TGGAATGTAAAGAAGTATGGAG | Scaffold-54 | 93962 | + |
| miR-n19-4 | TCAATACGGAGGATGAGGAAGAGCT | CTGTCATTTTCTCCGTCCTGGAAA | Scaffold-216 | 564739 | + |
| miR-n169 | GAAGGTCATATCTGTGTGGAATGTC | CAGTCTCCTACGCAGAACGGATATTAAA | Scaffold-1 | 2702632 | - |

| | | | | | |
|---|---|---|---|---|---|
| miR-n90b-2 | TGTATTCACCTTTTAGCACGTTCA | TGTGATGTGAGGTGAGTGCAAT | Scaffold-215 | 515477 | + |
| miR-n107c-1 | TGAGGTCTCGCAGAACTGAATT | TTCAGTCTGCAAAACCTCTAGA | Scaffold-1096 | 36194 | + |
| miR-34-1 | TGGCAGTGTGGTTAGCTGGTTGT | CGGCCACTTCCCACACTGCCCTC | Scaffold-317 | 185741 | - |
| miR-n52a-3 | CAGGTACCGTGTTTGATATTGTC | TAATATCATTCACGGTACTGCT | Scaffold-160 | 290628 | + |
| miR-n136a-1 | ATCATGAGGGGATTCGACGTTAACA | TTTGTTGGCCCCTCATAATGG | Scaffold-103 | 1582173 | + |
| miR-n195a-5 | AGCTGGCATACTTGTTTCCTATG | TAGGAACAATTATGCCGGTTTT | Scaffold-542 | 165843 | - |
| miR-n31 | AATGCTTTTACTGATTACTGAAAG | TCCTCATCAGTACAAGCAAACA | Scaffold-9 | 1077545 | + |
| miR-n203-4 | TGGTGGAGACGGGAAAAATTATA | TATTTTTCTCGTTTGCGCCAGA | Scaffold-542 | 205647 | - |
| miR-n210 | CACTCGCAGCCATTCCTTCCACCT | CAAGGAATGGCTGAGAGCGAAT | Scaffold-61 | 1776283 | - |
| miR-n39-2 | AAGTTTTAGTACATCATCATGCGAC | TTGGCATGGAATCTGGAATCATTGA | Scaffold-13 | 2359807 | + |
| miR-n192c-3 | TGGCAATGCAGGAGCTTTAATTG | ATAAAGCTTTTGCACTGTCAGA | Scaffold-71 | 141672 | + |
| miR-2b-1 | CACATCAAAGTGGCTGTGAAATG | TATCACAGCCAGCTTTGATGAGCGT | Scaffold-287 | 301896 | - |
| miR-n180a-1 | TTGTAGTCAACTTGCAGCACATT | TGTTATGTGAGATGAGTACATT | Scaffold-1096 | 42737 | + |
| miR-n121b-1 | ATTGGTTAATTGAGCTTTAGCG | TTAAAGCTTAGTTACCGATCA | Scaffold-531 | 85420 | + |
| miR-n40 | TGGTACCTTTCACAACAATGTC | ACATTGTGTGATAGTTACCATTCTT | Scaffold-215 | 733889 | - |
| miR-n189c-3 | TTGAACTTCATTCTCATTTCGTTTG | TGAAATGATAATGAAACTCAAGG | Scaffold-215 | 671338 | - |
| miR-n156 | TCTGATACACACCAAAGACCCG | TCTTTGGTATGTATTGGGCTATT | Scaffold-233 | 172195 | - |
| miR-n22 | TGAAGTGCTTTCTAGTACGGGCA | CCCGTACTTTAAAGCACTTCACC | Scaffold-1023 | 17008 | + |
| miR-34-2 | TGGCAGTGTGGTTAGCTGGTTGT | CGGCCACTTCCCACACTGCCCTC | Scaffold-527 | 122334 | - |
| miR-2788 | | CAATGCCCTTAGAAATCCCAA | Scaffold-759 | 84395 | - |
| miR-n121c | ATTGGTTAATTGAGCTTTAGCG | TTAAAGCTTTATTACCGATT | Scaffold-622 | 139289 | + |
| miR-n157 | TCTGGTCATGAGAAAATGAATT | TTCCTTTCTTTATGGCTGAAG | Scaffold-43 | 1979700 | + |
| miR-87-2 | CGCCTGATACTTGCTTCAAACCT | GTGAGCAAAGTTTCAGGTGTGT | Scaffold-42 | 930562 | + |
| miR-2796 | AGGGGTTTCTTTCGGCCTCCAG | GTAGGCCGGCGGAAACTACTTGC | Scaffold-30 | 2073397 | + |
| miR-n56a-3 | TGGTAAGTTCCACCACTATCTCA | AGATAGGGTGGTTGTTACCATTC | Scaffold-215 | 768640 | - |

| | | | | | |
|---|---|---|---|---|---|
| miR-n81a-1 | TGATGGAAGGGAGTTCCTCAAT | TGTGGAGCTCCTCTTCCATCAGA | Scaffold-15 | 7254094 | + |
| miR-n137b | TGGGGCCATGAGGGACTGAATTT | ATTTGTTCCTTATGGCCTCATGA | Scaffold-215 | 418010 | + |
| miR-2765b-1 | TGGTAACTTCACCACCGTTGGC | CAACGGTGCTGTAGTTCCTACA | Scaffold-161 | 770754 | + |
| miR-n63b-3 | TGTTGTTGGGAGACATGAAATG | TTTCATGTCTATGCCCAACAGCA | Scaffold-15 | 7267458 | + |
| miR-n13 | TGTGTAGATTCCGAATCCGAGTGAT | TTTTTTGTAATTCGAGATGGCGGCC | Scaffold-202 | 783947 | - |
| miR-n112 | TTTAGAATTCTAATCTAAAA | CGATTAGAGAAGAATTCAAAGC | Scaffold-47 | 914837 | + |
| miR-n2 | TGATTTCCATCAATTACAAGGATTG | TGTAGTTGATGGATTGATTGAAGAT | Scaffold-300 | 178268 | + |
| miR-283a-1 | CAATATCAGCCGGTAATTGCGA | CGAGATTGCCCACTGATATTCA | Scaffold-57 | 35286 | - |
| miR-n140-1 | TTTGGAGGGGACTTATTGCAT | TGCAATATTCCTTTTCCGATA | Scaffold-28 | 1826284 | + |
| miR-n111 | TGTATTAACTGACCTGAAGATT | TAGTCAGTTCAGTTGATA | Scaffold-43 | 1990631 | + |
| miR-n56b-1 | TGGTAACTTCCACCACTATCT | AGATAGGGTGGTTGTTATCATTCT | Scaffold-215 | 416067 | + |
| miR-n165-1 | CTTGCACCACAGAATTCACATTT | TGAGTGTTCTCTGACCATAAGGCTG | Scaffold-74 | 2416021 | - |
| miR-n155 | AAAATGACTGCCGAATCGGACT | TCCCGGTTCGAAAGTCATTCAG | Scaffold-21 | 2483115 | - |
| miR-n144b | TGGTGGAGACGGGATCAATTAT | TATTAATCTCGTTTCCACCAG | Scaffold-15 | 7235355 | + |
| miR-n174 | TGTGATGTTTTTGTGGGTCGTT | CGGCCCATAACGACATCATGTT | Scaffold-29 | 1947746 | - |
| miR-n126b-2 | CAGGAAGCGGGGGTGGAAAT | CTTCCACCTCCTCTTCCTCGAA | Scaffold-215 | 492848 | + |
| miR-n32b | GAAGTCATCTCAGCATTATATGC | AGTGATGCTGTGATGACTTCACT | Scaffold-205 | 690974 | - |
| miR-n165-2 | CTTGCACCACAGAATTCACATTT | TGAGTGTTCTCTGACCATAAGGCTG | Scaffold-74 | 2408581 | - |
| miR-n57-2 | CGAGATCATAATGAAAGTTACGCT | TGGGCTTTCAGTATGATCTGTC | Scaffold-215 | 693371 | - |
| miR-n202a-8 | TTTAAAGGGGCCTCACAGTAACG | TTACTGTGCGGCTACTTTATCT | Scaffold-215 | 770391 | - |
| miR-n192a-2 | TGGCAATGCAGGAGCTTTAATTG | ATGAAGCTTTTGCACTGTCAGA | Scaffold-71 | 140131 | + |
| miR-n202a-7 | TTTAAAGGGGCCTCACAGTAACG | TTACTGTGCGGCTACTTTATCT | Scaffold-215 | 729537 | - |
| miR-iab-4 | ACGTATACTGAATGTATCCTGA | TATACCTTCAGTATACGTAACA | Scaffold-72 | 8695284 | + |
| miR-283a-2 | CAATATCAGCCGGTAATTGCGA | CGAGATTGCCCACTGATATTCA | Scaffold-57 | 37243 | - |
| miR-n188b | TGGGGTCATGCAGAGCTGGAT | TCACAGCCTGCATAATCCCAAG | Scaffold-215 | 673881 | - |

| | | | | | |
|---|---|---|---|---|---|
| miR-n194e | TGTATGGAGGGTTCTTCTCAAT | TGAGGAGTTCCCCCCATTCATA | Scaffold-15 | 7288695 | + |
| miR-n194b-2 | TAGTATGGAGGGCTCTTCTCAAT | TGAGGAGTTCCCCCATATTCAT | Scaffold-15 | 7235668 | + |
| miR-n167 | TGCTCCATCGTAGCTTTGAGA | TCAAACAGTCTGTGATGGAAATAGC | Scaffold-29 | 2622701 | - |
| miR-n37-3 | TGTACCAGCCGTCCTGGTGGAT | TATCCAGTGCAGTTGGTACAAT | Scaffold-215 | 511156 | + |
| miR-n90 | GTATTCACCTTTCAGCACATTC | TGTGATGTTAGGTGAGTGCATT | Scaffold-215 | 685848 | - |
| miR-n38c | TGTATCAACCGACCTGTAGATT | TTTAAAGGGCAGTTGATACAAT | Scaffold-215 | 702712 | - |
| miR-n181 | TCGTGGCTCCCCAGGTATTAATT | TTAATGACTGTGGAGCTGCAAT | Scaffold-215 | 627750 | - |
| miR-n178-1 | AGGTGGTTTTAGCCCGGTTCGA | GCACCGGGTGAGAATCGCCTTA | Scaffold-531 | 81288 | + |
| miR-n37-1 | TGTACCAGCCGTCCTGGTGGAT | TCCCCAGTGCAGTTGATACAAT | Scaffold-215 | 455483 | + |
| miR-3049-2 | TCGGGAAGGCAGTTGCGGCGGATT | TCCGTCCAACTCCTTTCCGTCT | Scaffold-349 | 196342 | + |
| miR-10 | TACCCTGTAGATCCGAATTTGT | CAAATTCGGTTCTAGAGAGGTTT | Scaffold-72 | 7151097 | - |
| miR-n100-2 | TGTTGTTGGGAGACCAAAATTGT | TAATTTGGTCTACTCAACAGCACA | Scaffold-828 | 61826 | - |
| miR-n195a-4 | AGCTGGCATACTTGTTTCCTATG | TAGGAACAATTATGCCGGTTTT | Scaffold-15 | 7278853 | + |
| miR-9a-2 | TCTTTGGTTATGTAGCTGTATGA | ATAAAGCTATATTACCAAAGCA | Scaffold-113 | 387984 | + |
| miR-n146-1 | TGAGTAGACACGCTTTAGCAATAC | TGTAAGAGCTTGTCTTCTCAGC | Scaffold-797 | 102879 | - |
| miR-281 | AAGAGAGCTATCCGTCGACAGT | TGTCATGGAGTTGCTCTCTT | Scaffold-15 | 2099415 | - |
| miR-n75-3 | GCTGGGACTCTTGGGCCTTATG | TAAGGTACATTAGTCTCAGTGT | Scaffold-24 | 95808 | + |
| miR-12-1 | AGAGTATTATATCAGGTACTGGT | | Scaffold-312 | 7337 | + |
| miR-1000 | ATATTGTCCTGTCACAGCAGT | CTGCTGTATCAGGACATGCCC | Scaffold-335 | 174697 | + |
| miR-n63 | TGTTGTTGGGAGACATGAAATG | TTTCATGTCCATGCCCAACAGCA | Scaffold-542 | 195555 | - |
| miR-n59-2 | TGATGGTAATTGCCTGGTCGATGT | TCACCGGGCACTTACTACCAACT | Scaffold-531 | 87791 | + |
| miR-n194g-2 | TTGTATGGAGGGCTCTTCTCA | TGAGGAGTTCCCCCATATTCAT | Scaffold-542 | 192740 | - |
| miR-n173b-1 | GAAGTCATCTCAGTATTATATG | AGTGATGCTGTGATGACTTCA | Scaffold-24 | 131576 | + |
| miR-n82b | GGGTAGTGTTTCTGATATTGTC | TAATATCATTGACACTACTTTT | Scaffold-160 | 177302 | + |
| miR-n67-2 | AGTAATTATCGCGTCAATCT | AGATTGTGTGATAGTTACCATTCA | Scaffold-215 | 576503 | + |

| | | | | | |
|---|---|---|---|---|---|
| miR-n202a-6 | TTTAAAGGGGCCTCACAGTAACG | TTACTGTGCGGCTACTTTATCT | Scaffold-215 | 704597 | - |
| miR-7 | TGGAAGACTAGTGATTTTGTTGTT | CAATAAGTCTCTAGTCATCCTA | Scaffold-31 | 2497271 | + |
| miR-747-2 | TAATCTCATGTGGTAATGATACA | TATCATTATCAAATGGGATT | Scaffold-57 | 37371 | - |
| miR-n196a-6 | CGAGGAACATTTTCTGGTCAT | TGACTAGATACTGTTCCTCAGA | Scaffold-215 | 704954 | - |
| miR-305 | ATTGTACTTCATCAGGTGCT | CGGCACCTGATAGAGTGCAATTC | Scaffold-77 | 261828 | + |
| miR-n178-2 | AGGTGGTTTTAGCCCGGTTCGA | GCACCGGGTGAGAATCGCCTTA | Scaffold-622 | 135847 | + |
| miR-n98a-2 | AGGAAAGAGAAAGAGGGCAAATG | TTGATCATTTTCTCTTTTCTGT | Scaffold-7 | 1446725 | - |
| miR-965 | CGGGAAAGGTTATAGCGATTATG | TAAGCGTATAGCTTTTCCCCT | Scaffold-181 | 494214 | - |
| miR-2765a-1 | TGGTAACTCCACACCACCGTTGGC | CAATGGTTGTGGAGTTCCCCTT | Scaffold-12 | 847776 | + |
| miR-13a-1 | CGTCAAATTGGTTGTGAGTTATG | TATCACAGCCACTTTGATGAAC | Scaffold-287 | 306736 | - |
| miR-n180a-2 | TTGTAGTCAACTTGCAGCACATT | TGTTATGTGAGATGAGTACATT | Scaffold-215 | 596950 | - |
| miR-252b-1 | TAAGTAGTAGTGCCGTAGCGA | | Scaffold-225 | 289577 | - |
| miR-n177-1 | AAGGAACTACTGGTGTGCATAT | TATCACAGCCGTAATTCCTGTG | Scaffold-531 | 81162 | + |
| miR-n72 | GGGGCTGAAGCTGGGATTGTC | CATCACAGTTTCAGCCCCGAGA | Scaffold-442 | 90832 | - |
| miR-n189c-2 | TTGAACTTCATTCTCATTTCGTTTG | TGAAATGATAATGAAACTCAAGG | Scaffold-215 | 668380 | - |
| miR-n128-3 | TGAGGAACATTTTCTGGTCATA | TGACTAGATTCTGTTCCTCAGA | Scaffold-215 | 568299 | + |
| miR-n3-4 | ATGGAGTCCGAAGTCACAGAACATT | TTCATGTGGCGGAATTGACTTCA | Scaffold-1154 | 28128 | - |
| miR-n107a-1 | TGAGGTCTCGCAGTACTAAATT | TTTAGTCTGCAAAACCTCAAGATA | Scaffold-215 | 487263 | + |
| miR-n27 | AGTCGAACAGCTGTAGCCATTGT | TTGGCTACAGATGTTCGACGGGT | Scaffold-409 | 121408 | - |
| miR-n65-2 | TTGGAACTATTGTGTCAATCTC | TCATTGCATGATAGTTACCATTC | Scaffold-43 | 1990806 | + |
| miR-n52a-2 | CAGGTACCGTGTTTGATATTGTC | TAATATCATTCACGGTACTGCT | Scaffold-160 | 261727 | + |
| miR-n70 | ACTGGCTGGTGATGATGCTG | CATCGGACCCAAGTCAGTTCGT | Scaffold-414 | 192615 | - |
| miR-317-2 | AGGGAACCACCCTGGGTTCACT | TGAACACAGCTGGTGGTATCT | Scaffold-527 | 151500 | - |
| miR-n66 | TGCTGTTGGGAGACATTTAATG | TTAATGCCTAGCCAACAGCACA | Scaffold-205 | 693240 | - |
| miR-n30 | TATCCATGAACTTTCAATACT | AACAAGTTCATGGTACATTG | Scaffold-175 | 888843 | + |

| | | | | | |
|---|---|---|---|---|---|
| miR-n28 | TTGCAGATCGCTTGTAGACTGTT | AACACTCACCAGCGATCTGCCA | Scaffold-94 | 562026 | - |
| miR-n125-2 | CTTGGGAGAGTTTTATTGTATG | TACGTAAAGCCCCTCCCAAGGA | Scaffold-24 | 121923 | + |
| miR-n78a-1 | AGTTGGACAGTTCAAGATTGTTG | CCAACCTTGGCTTTCCGACTGTAG | Scaffold-7 | 1441464 | - |
| miR-n202c | TTTAAAGGGGCCTCACAGTAACA | TTACTGTGCGGCTACTTTATCT | Scaffold-215 | 746941 | - |
| miR-275 | CGTGCTGCATCAGGGACTTAGTGAC | TCAGGTACCTGAAGTAGCGCGCG | Scaffold-77 | 259466 | + |
| miR-n103 | CCTGAGAAGATACGTCGTTACA | TAACCGCGTGTCTTCTTAGCTT | Scaffold-383 | 134399 | - |
| miR-n194f-1 | TAGTATGGAGGGTTCTTCTCAAT | TGAGGAGTTCCCCCATATTCAT | Scaffold-542 | 172630 | - |
| miR-n105c | CTTCTCATGATCCCGGTGATACA | TACACTGGGATTATGAGATTTA | Scaffold-160 | 289882 | + |
| miR-n196a-5 | CGAGGAACATTTTCTGGTCAT | TGACTAGATACTGTTCCTCAGA | Scaffold-215 | 621920 | - |
| miR-193-1 | TCGGGACTTGGTGGTATAGTTGG | TACTGGCCTGCTAAGTCCCAAG | Scaffold-1472 | 20957 | + |
| miR-n188c | TGGGATCATGCAGAGCTGGTTTTGA | TCACAGCCTCCATAATCCCAAG | Scaffold-215 | 706397 | - |
| miR-n198c | TGAGGTCATGCAGAACTGAATT | TTTGGTTCCTCATGGCCTCACGA | Scaffold-215 | 731619 | - |
| miR-n19-3 | TCAATACGGAGGATGAGGAAGAGCT | CTGTCATTTTCTCCGTCCTGGAAA | Scaffold-16 | 889558 | + |
| miR-998 | AGCTGAATGTTGTGGTGTGGCA | TAGCACCATGGCATTCAGCTT | Scaffold-442 | 89242 | - |
| miR-n37-2 | TGTACCAGCCGTCCTGGTGGAT | TCCCCAGTGCAGTTGATACAAT | Scaffold-215 | 478333 | + |
| miR-n43a | TGAGATCATCATGTTGATTACT | TATTCTCATGATTGATCTCAAG | Scaffold-160 | 227344 | + |
| miR-n56a-2 | TGGTAAGTTCCACCACTATCTCA | AGATAGGGTGGTTGTTATCATTCT | Scaffold-215 | 702538 | - |
| miR-n192c-4 | TGGCAATGCAGGAGCTTTAATTG | ATAAAGCTTTTGCACTGTCAGA | Scaffold-71 | 143665 | + |
| miR-n49 | CAGGTACTGTTCTTGATATTGTC | TAATATCATTAACAGTACTGTT | Scaffold-160 | 188368 | + |
| miR-n173b-2 | GAAGTCATCTCAGTATTATATG | AGTGATGCTGTGATGACTTCA | Scaffold-828 | 56864 | - |
| miR-n53 | TGGTAACTCTCACATCAATGTCA | GCATGCTGTGATAGTTACCATTCA | Scaffold-1096 | 39847 | + |
| miR-n194b-3 | TAGTATGGAGGGCTCTTCTCAAT | TGAGGAGTTCCCCCATATTCAT | Scaffold-15 | 7260562 | + |
| miR-n183-1 | CGGTCTGTCAAGAGTATTTCTATG | TAGATTTCTCTTGACAGGCTTGA | Scaffold-1436 | 1554 | + |
| miR-n144a-2 | TGGTGGAGACGGGATTAATTATA | TATTAATCTCGTTTGCACCAG | Scaffold-15 | 7288537 | + |
| miR-n144c | TGGTGGAGACGGGATTAATTATATT | TATTAATCTTGTTTCCACCAGA | Scaffold-542 | 172475 | - |

| | | | | | |
|---|---|---|---|---|---|
| miR-n102-4 | CTTCTCATGTCCATGCTGATACA | TAGCACATGCTCATGAGAAG | Scaffold-215 | 646300 | - |
| miR-n92 | TGATGGAAGGGAGTTCCTT | TAGAGAGCTCTTCTTCCATCAGA | Scaffold-542 | 164172 | - |
| miR-n194d | TGTATGGCGGGTTCTTCTCAAT | TGAGGAGTTCCCCCCATTCATA | Scaffold-15 | 7269283 | + |
| miR-n208 | TGGTGATTCAATGATGACTGTG | CACGGTGTTATTGAGAGAGCTG | Scaffold-11 | 2398064 | + |
| miR-n36-4 | TAGGTGCTGTTTTTGATATTGTC | TAATATGAAAAATATCCCTGCTTT | Scaffold-160 | 222386 | - |
| miR-n18 | AAACAGTCGATAATATACAGCT | TTGTGTATTATACAACTGTGACA | Scaffold-132 | 891789 | - |
