# Supplementary material for: Characterization and comparative analysis of microRNAs in the rice pest Sogatella furcifera
Source: PLoS One. 2018 Sep 24;13(9):e0204517. doi: 10.1371/journal.pone.0204517 (PMC6152972; doi:10.1371/journal.pone.0204517)
Supplement: S3 Table — (PDF) [file pone.0204517.s006.pdf]

S3 Table. Differentially expressed miRNA in male and female adults

Up-regulated in Female	logFC	P-value	Up-regulated in Male	logFC	P-value
miR-275-3p	0.47	8.22E-04	miR-1000-5p	-0.63	5.11E-04
miR-2765a-1-5p	1.20	9.21E-10	miR-10-5p	-0.79	3.20E-10
miR-2765a-2-5p	1.00	7.31E-12	miR-124-3p	-0.59	2.52E-03
miR-2765b-1-5p	0.91	1.81E-08	miR-125-5p	-0.65	3.23E-05
miR-305-5p	0.92	6.42E-09	miR-133-3p	-0.51	1.86E-04
miR-306-5p	0.78	5.59E-08	miR-137-3p	-0.90	1.13E-09
miR-33-3p	1.32	3.34E-06	miR-1-3p	-0.40	9.31E-04
miR-71-1-5p	0.70	1.34E-06	miR-14-3p	-0.59	8.25E-05
miR-8-3p	0.66	1.05E-06	miR-184a-3p	-0.58	4.67E-06
miR-998-3p	0.67	1.67E-05	miR-184b-3p	-0.68	5.79E-07
miR-9a-1-5p	0.78	4.42E-08	miR-210-1-3p	-0.82	8.12E-08
miR-9b-1-5p	0.70	1.43E-07	miR-210-1-5p	-1.05	7.22E-12
miR-9c-5p	1.19	1.38E-18	miR-219-3p	-1.42	3.82E-08
miR-iab-4-5p	0.91	1.36E-07	miR-252a-1-5p	-1.07	1.94E-13
miR-n105b-1-3p	3.29	2.87E-08	miR-263a-1-5p	-1.31	3.79E-20
miR-n108-3p	7.24	7.27E-187	miR-263b-5p	-1.33	2.20E-21
miR-n109-5p	7.36	9.77E-201	miR-277-1-3p	-0.53	1.29E-04
miR-n113-1-3p	3.75	4.42E-13	miR-278-3p	-0.61	4.72E-05
miR-n113-1-5p	3.45	2.72E-18	miR-2796-3p	-0.81	4.86E-09
miR-n11-5p	2.88	1.18E-03	miR-29-3p	-0.67	8.54E-05
miR-n121-3p	6.36	6.65E-91	miR-3049-1-5p	-1.17	7.39E-11
miR-n121b-1-3p	6.36	3.11E-92	miR-315-5p	-0.48	8.96E-04
miR-n121c-3p	5.35	3.73E-54	miR-316-5p	-0.90	4.18E-11
miR-n122-3p	3.72	5.84E-05	miR-317-1-3p	-0.46	2.29E-04
miR-n132-1-3p	5.35	9.73E-55	miR-34-1-5p	-0.89	2.12E-07
miR-n132-1-5p	3.76	1.61E-36	miR-7-5p	-0.92	7.85E-11
miR-n136a-1-3p	0.59	2.21E-03	miR-87-1-3p	-0.43	2.32E-03
miR-n141-1-3p	1.97	5.44E-06	miR-927-1-5p	-1.00	4.87E-11
miR-n144a-1-3p	2.93	1.32E-10	miR-929-5p	-0.68	1.16E-03
miR-n144b-3p	3.66	7.19E-14	miR-92b-3p	-0.57	4.09E-04
miR-n145-3p	3.00	7.58E-04	miR-932-5p	-0.89	1.50E-07
miR-n147-3p	1.28	1.86E-03	miR-971-1-5p	-1.27	4.94E-14
miR-n166-5p	1.25	2.65E-03	miR-981-3p	-1.12	3.28E-11
miR-n168-5p	4.56	1.32E-10	miR-981-5p	-1.33	7.99E-12
miR-n169-5p	5.84	3.50E-06	miR-993-3p	-1.26	4.70E-17
miR-n171-5p	5.68	5.51E-05	miR-n102-3-3p	-1.72	1.55E-04
miR-n172-1-5p	5.79	8.29E-17	miR-n104-5p	-4.79	2.23E-19
miR-n174-5p	13.12	0.00E+00	miR-n106-3p	-0.88	6.81E-05
miR-n175-1-3p	3.53	7.48E-19	miR-n107a-1-3p	-2.05	3.95E-13
miR-n176-5p	0.57	1.85E-04	miR-n107b-5p	-2.93	2.82E-36
miR-n177-1-5p	2.95	8.23E-06	miR-n107c-1-3p	-2.43	4.91E-35
miR-n178-1-3p	5.02	2.12E-19	miR-n107d-1-3p	-2.11	3.22E-15

miR-n182-3p	3.86	3.76E-05	miR-n112-3p	-0.75	1.76E-04
miR-n183-5-3p	1.84	2.09E-17	miR-n115a-1-3p	-1.25	4.29E-04
miR-n184-5p	2.81	8.43E-05	miR-n115a-1-5p	-1.81	1.61E-06
miR-n186-3p	5.33	7.55E-26	miR-n115a-3-3p	-1.25	4.41E-04
miR-n187-3p	5.45	6.14E-05	miR-n116-3p	-2.43	2.22E-03
miR-n187-5p	4.94	1.96E-03	miR-n124-3p	-1.56	3.78E-04
miR-n192a-1-3p	2.56	2.05E-14	miR-n127-3-5p	-3.59	1.76E-11
miR-n192b-3-3p	2.38	2.50E-31	miR-n128-1-3p	-2.17	4.94E-17
miR-n192c-2-3p	2.36	9.90E-36	miR-n130-1-5p	-3.20	4.15E-35
miR-n194a-5p	2.03	9.27E-04	miR-n137b-3p	-2.08	1.54E-05
miR-n194b-1-5p	2.04	1.21E-11	miR-n137e-3p	-2.58	3.62E-04
miR-n194c-1-5p	2.96	1.64E-08	miR-n138-5p	-2.94	6.45E-22
miR-n194e-5p	2.23	2.26E-05	miR-n149-1-5p	-2.49	5.17E-09
miR-n194f-1-5p	2.11	5.45E-04	miR-n170-3p	-0.43	3.81E-03
miR-n194g-1-5p	1.41	6.55E-04	miR-n173a-3p	-2.18	8.46E-04
miR-n195a-1-3p	1.22	2.88E-03	miR-n173b-1-3p	-1.71	1.99E-03
miR-n197-5p	3.29	7.70E-05	miR-n180b-5p	-1.88	2.98E-04
miR-n203-1-3p	1.97	4.40E-07	miR-n181-3p	-1.75	1.15E-19
miR-n207-3p	4.14	4.10E-08	miR-n188b-3p	-3.62	1.95E-04
miR-n208-5p	5.56	3.08E-05	miR-n189c-1-5p	-2.39	1.51E-04
miR-n209-1-3p	6.75	2.04E-26	miR-n196a-1-3p	-0.98	1.73E-05
miR-n33-3p	0.80	1.08E-03	miR-n196b-5p	-2.86	3.10E-12
miR-n36-1-5p	2.49	4.74E-22	miR-n198a-5p	-3.59	1.74E-11
miR-n43e-3p	3.07	2.29E-04	miR-n198b-1-3p	-2.05	6.29E-06
miR-n51-5p	1.73	2.02E-04	miR-n198b-1-5p	-2.94	9.06E-22
miR-n52b-1-3p	4.22	1.06E-06	miR-n202a-1-3p	-1.71	1.93E-05
miR-n58-5p	14.00	0.00E+00	miR-n202a-1-5p	-1.81	9.99E-06
miR-n59-1-3p	2.37	9.61E-41	miR-n204a-1-3p	-3.07	2.18E-06
miR-n63-3p	2.57	3.18E-08	miR-n206-3p	-4.95	3.83E-22
miR-n63-5p	2.09	2.64E-08	miR-n32b-3p	-1.71	2.10E-03
miR-n63b-1-3p	2.17	8.40E-07	miR-n38b-1-3p	-1.47	6.06E-04
miR-n72-3p	0.60	9.37E-04	miR-n38b-1-5p	-3.92	1.83E-19
miR-n81a-1-3p	2.99	1.45E-05	miR-n38e-5p	-3.92	2.13E-19
miR-n81b-5p	4.67	2.13E-07	miR-n53-5p	-1.80	4.16E-05
miR-n83-3p	4.64	2.46E-16	miR-n56a-1-5p	-2.87	3.17E-23
miR-n88-3p	3.10	1.65E-21	miR-n56b-1-5p	-2.22	1.00E-11
			miR-n76-5p	-1.09	2.36E-03
			miR-n77a-5p	-2.08	5.39E-15
			miR-n78a-3-3p	-1.90	1.36E-05
			miR-n78b-5p	-2.61	1.40E-09
			miR-n80-1-3p	-1.76	1.98E-03
			miR-n90-3p	-2.15	2.26E-09
