# Supplementary material for: Characterization and comparative analysis of microRNAs in the rice pest Sogatella furcifera
Source: PLoS One. 2018 Sep 24;13(9):e0204517. doi: 10.1371/journal.pone.0204517 (PMC6152972; doi:10.1371/journal.pone.0204517)
Supplement: S4 Table — (PDF) [file pone.0204517.s007.pdf]

S4 Table. Primers used in RT-qPCR for validation of the miRNA genes

Target gene	Primer sequence	
SFU-348.23	F	CGTGAAACTTTTGATTTCAT
	R	TCTTCGGTATTCGTGTTCTT
SFU-40.81	F	CAAAAGTGACGACAGTGCCA
	R	TCGCCATTGGTAAGATCGGA
SFU-162.31	F	GATAGGGGAGAACGGGACAG
	R	TGGGACTCCTACTCCTCGAA
SFU-17.151	F	TCGATGGAGAAGGGAAGCTC
	R	TTTGGCAAGGAACAGTCTGC
SFU-RPL4	F	AATCATGGGGAACTGGTCGT
	R	GCGGAGACCATAGCATACCT
